# A Study on Hyperspectral Non-Destructive Testing of Mechanical Damage in Yali Pears Using Linear Dimension Reduction and a Lightweight CNN

**DOI:** 10.3390/foods15173068

**Published:** 2026-08-29

**Authors:** Chao Ma, Ling Zhao, Yaning Chang, Junjie Ma, Fenglei Wang, Jun Qian, Huimin Fang

**Affiliations:** 1 School of Mechanical and Automotive Engineering (School of Precision Manufacturing), Liaocheng University, Liaocheng 252059, China; machao0085@163.com (C.M.); 13355123020@163.com (Y.C.); 15266822696@163.com (J.M.); wangfenglei@lcu.edu.cn (F.W.); qianjun@lcu.edu.cn (J.Q.); 2 School of Agricultural Engineering, Jiangsu University, Zhenjiang 212013, China; fanghuimin@ujs.edu.cn

**Keywords:** hyperspectral imaging, regularised linear discriminant analysis, lightweight convolutional neural networks, damage to Yali pears, non-destructive testing

## Abstract

To enable rapid, non-destructive identification of damage to Yali pears, this study proposes a detection method that integrates short-wave infrared hyperspectral imaging (1000–2500 nm), regularised linear discriminant analysis (R-LDA) and a lightweight convolutional neural network (CNN). The experiments utilised 180 Yali pears (60 each of healthy, with Mechanical scratch and Compression damage specimens) as training samples, whilst a further 300 independent fruits (150 healthy and 150 damaged) were used for fruit-level sorting validation. Following pre-processing using Principal Component Analysis (PCA) to eliminate multicollinearity, the classification performance of three feature extraction strategies—PCA, Independent Component Analysis (ICA) and R-LDA—was compared when combined with the same lightweight CNN. The results indicate that R-LDA’s Fisher discrimination criterion (5.0275) and separation index (2.3543) were both superior to those of PCA and ICA, and its dimension-reduced features exhibited stronger inter-class separability. In pixel-level testing, the R-LDA + lightweight CNN achieved recognition accuracies of 98.60 per cent and 99.32 per cent for Compression damage and background, respectively, and 93.54 per cent and 96.33 per cent for Mechanical scratch and intact tissue, respectively. In a validation study involving the sorting of 300 independent fruits, this method achieved a recall rate of 100.00% for damaged fruits (zero false negatives), with precision and F1 scores of 97.00% and 97.09% respectively, both of which outperformed PCA combined with a lightweight CNN and ICA combined with a lightweight CNN. The above results indicate that the combination of R-LDA discriminant dimensionality reduction and a lightweight CNN can effectively reduce redundancy in hyperspectral data whilst maintaining a high damage detection rate, thereby providing a viable solution for the rapid, non-destructive detection of post-harvest damage in Yali pears.

## 1. Introduction

The Yali pear is a common and important fruit for ensuring the supply of fruit and vegetables and increasing farmers’ incomes; however, mechanical forces during harvesting, transport and storage can easily cause damage, with mechanical scratch and Compression damage being the most typical examples [1]. Mechanical scratch manifests as ruptures or linear wounds in the fruit skin, whilst Compression damage involves damage to the internal cellular structure whilst the epidermis remains intact. In the early stages, compression damage is not readily apparent; manual sorting relies on visual inspection and experience, making it difficult to identify such damage consistently, objectively and efficiently. This is particularly problematic at high sorting speeds or when initial symptoms are subtle, leading to a higher incidence of missed or misidentified damage. Consequently, damaged fruit is likely to be mixed with healthy batches, thereby reducing storage quality, shortening shelf life and resulting in economic losses [2]. Therefore, the development of rapid, accurate and non-destructive detection methods for mechanical damage in Yali pears is of great significance for improving post-harvest sorting efficiency and safeguarding the commercial value of the fruit.

To overcome the limitations of manual sorting, non-destructive testing technologies—which can obtain information on a sample’s external morphology, internal structure or physicochemical properties without damaging it—have become a key area of development in agricultural product quality testing [3]. Among these, multispectral and hyperspectral imaging technologies combine image and spectral information to enable the extraction and identification of quality characteristics and defect information in agricultural products, aided by machine learning methods [4]. Unlike conventional machine vision, which can only capture surface colour and shape features, hyperspectral imaging technology can simultaneously acquire spatial image information and spectral response data across a continuous wavelength range. Characterized by the integration of ‘image and spectrum’, it captures changes in fruit tissue structure and physicochemical properties by analyzing differences in reflection and absorption at different wavelengths. This enables the identification of early-stage damage and internal quality changes that are difficult to detect with the naked eye, demonstrating significant application potential in fruit damage detection and internal quality assessment [5].

In recent years, the application of hyperspectral imaging technology in the non-destructive testing of fruit and vegetables has been extensively validated. Ekramirad et al. employed near-infrared hyperspectral imaging combined with a gradient tree boosting algorithm to detect apple codling moth infestation areas at the pixel level, achieving a validation accuracy of 97.4 per cent, demonstrating that hyperspectral imaging combined with machine learning methods can effectively identify surface defects on fruit [6]. Addressing the issue of early bruise detection in blueberries, Sun et al. combined hyperspectral reflectance imaging with band ratio analysis and an improved multi-threshold segmentation algorithm, achieving the identification and quantification of bruised tissue within 30 min of injury, with an average intersection-to-union ratio of 70.1 per cent, suggesting that hyperspectral imaging offers certain advantages in the detection of early potential damage [7]. Liu et al. utilised hyperspectral imaging combined with chemometric methods to classify samples of fresh jujubes from different varieties and stages of ripeness after drying, and to predict quality indicators such as soluble solids content and moisture content, thereby validating the application potential of hyperspectral technology in the evaluation of processed fruit quality [8]. Wang et al. addressed the issue of spectral interference caused by differences in variety and peel colour during the detection of soluble solids content in multiple peach varieties. They constructed a SIFNet model that fuses spectral information with image features, achieving a coefficient of determination of 0.8235, demonstrating that the fusion of spatial image information with spectral information helps to improve the stability and generalisation ability of internal fruit quality detection [9]. The aforementioned studies collectively demonstrate that hyperspectral imaging possesses the ability to capture both surface and internal damage characteristics in fruit; however, its recognition performance is highly dependent on subsequent feature extraction and dimension reduction strategies.

Although hyperspectral imaging has been widely applied in the non-destructive testing of fruit and vegetables, the simultaneous identification of Mechanical scratch and Compression damage in Yali pears still poses certain challenges. Hyperspectral data often comprises hundreds of contiguous bands, characterised by high dimensionality and significant redundancy; direct modelling not only increases the computational burden but may also introduce noise unrelated to classification. Existing research has pointed out that when hyperspectral imaging is combined with methods such as autoencoders, key issues such as high-dimensional data redundancy, feature compression and model parameter optimisation still need to be addressed [10]. Therefore, prior to feeding hyperspectral data into a classifier, selecting a dimension-reduction strategy that can effectively compress dimensions whilst retaining damage-discriminating information is a crucial step in achieving efficient and accurate recognition.

Among commonly used linear dimension-reduction methods, PCA (Principal Component Analysis) achieves unsupervised dimension reduction by identifying the orthogonal directions with the greatest variance, retaining only the global variance structure; ICA (Independent Component Analysis) assumes that the source signals are statistically independent and extracts non-Gaussian independent components, but likewise does not utilise class label information. For the task of distinguishing between Mechanical scratch and Compression damage on Yali pears, the spectral differences between damage categories are often subtle; the directions or components retained by PCA and ICA are not necessarily directly relevant to damage discrimination, and they have inherent limitations when distinguishing between damage categories with highly similar spectral features. In contrast, R-LDA (Regularised Linear Discriminant Analysis, R-LDA) directly learns the projection directions most relevant to class discrimination by maximising inter-class divergence and minimising intra-class divergence, compressing high-dimensional spectral data into low-dimensional features with greater discriminative power, making it better suited to the requirements of this study. With regard to model selection, traditional machine learning methods have limited capacity for the automatic extraction of complex spatio-spectral joint features, and their performance is easily influenced by feature selection methods and sample variations [11]. Although deep learning models such as CNNs possess strong hierarchical feature learning capabilities, their complex network parameters and high computational demands are not conducive to real-time sorting deployment [12]. Therefore, this study adopts a lightweight Convolutional Neural Network (CNN) as the classifier, which maintains the ability to extract spatiospectral joint features whilst controlling the number of model parameters and inference time, thereby meeting the real-time requirements of online sorting.

Addressing the issues associated with short-wave infrared (SWIR) hyperspectral data—such as high spectral dimensionality, severe information redundancy, and pronounced multicollinearity among variables—as well as the large number of parameters in traditional detection models, which make it difficult to meet the real-time sorting requirements for fruit, this paper proposes a method for distinguishing damage in duck pears based on SWIR hyperspectral imaging combined with Regularised Linear Discriminant Analysis (Regularised Linear Discriminant Analysis, R-LDA) and a lightweight Convolutional Neural Network (CNN). Firstly, Principal Component Analysis (PCA) is employed for spectral data pre-processing, effectively eliminating multicollinearity between bands and compressing the raw spectra into orthogonal and uncorrelated principal components. The PCA transformation matrix is fitted using only the training set data, thereby laying a reliable data foundation for subsequent discriminant feature extraction. On this basis, the study compared the recognition performance of three feature extraction strategies—PCA (Principal Component Analysis), ICA (Independent Component Analysis) and R-LDA—when combined with the same lightweight network. By fully utilising the advantage of R-LDA in enhancing class discrimination through label information, the optimal combination model of R-LDA dimensionality reduction coupled with a lightweight CNN was selected. This method not only resolves the computational instability issues associated with the direct application of traditional LDA to raw spectra but also significantly reduces the computational overhead of the model whilst ensuring recognition accuracy. It provides an efficient and feasible technical approach for the rapid and accurate identification of different types of mechanical damage in Yali pears, as well as for industrialised online sorting.

## 2. Materials and Methods

### 2.1. Overall Technical Approach

**Figure 1:**
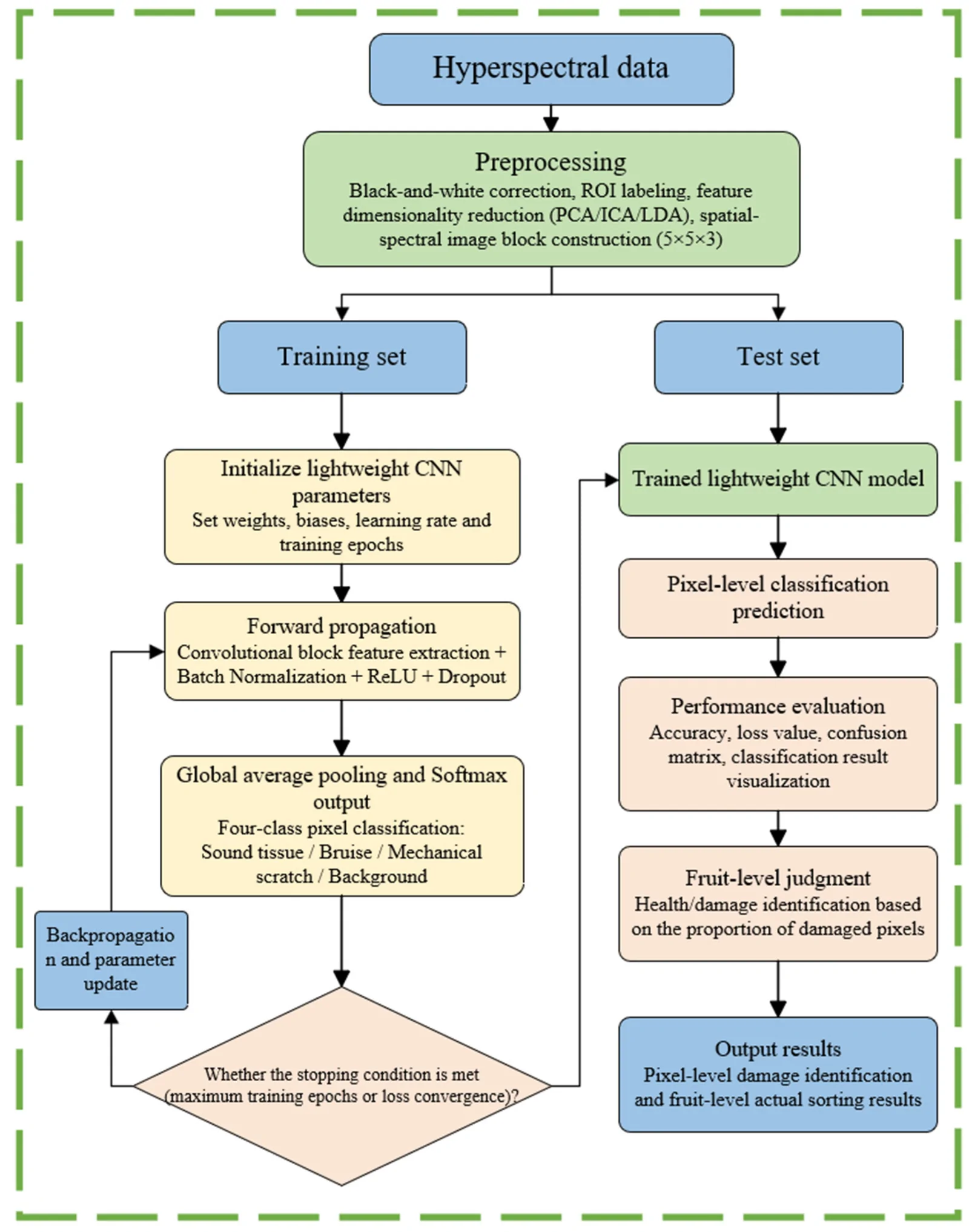
A Technical Approach to Non-Destructive Hyperspectral Identification of Damage in Yali Pears.

The non-destructive testing workflow for damage in Yali pears proposed in this study is shown in Figure 1, comprising three stages: data pre-processing, model training, and testing and evaluation. During the pre-processing stage, the raw hyperspectral images undergo greyscale correction to eliminate noise; after annotating Regions of Interest (ROIs) to filter out valid regions, spatial–spectral image patches are constructed based on a unified PCA pre-processing method fitted solely to the training set, and these are fed into a lightweight CNN. The Fisher discrimination criterion, separation index and computation time for each method are also evaluated. During the model training stage, the lightweight CNN takes the pre-processed image patches as input. Following convolutional feature extraction, batch normalisation, ReLU activation and Dropout regularisation, the network performs a forward pass; parameters are then iteratively updated via backpropagation based on prediction errors until convergence is achieved. During the testing phase, the fully trained model performs pixel-level predictions on the test set. Performance is evaluated using accuracy, confusion matrices and classification visualisations, and fruit-level binary classification (intact/damaged) is achieved via a voting mechanism based on the proportion of damaged pixels per fruit. Finally, the fruit-level sorting performance of the three feature extraction strategies (PCA, ICA and R-LDA) was validated using 300 independent fruit samples, providing a technical reference for non-destructive post-harvest inspection of Yali pears.

### 2.2. Hyperspectral Imaging Technology and System Configuration

Hyperspectral imaging combines spectral analysis with image recognition technology, enabling the simultaneous acquisition of a sample’s spectral information and its spatial location across a continuous wavelength range, thereby generating a three-dimensional data cube. This technology can capture subtle spectral differences that are difficult to discern with conventional visual inspection and has been widely applied to agricultural product inspection tasks such as fruit damage, ripeness, external defects and internal quality [13]. Short-wave infrared imaging can acquire spectral information related to changes in moisture content, tissue structure and chemical composition across a relatively broad wavelength range, making it particularly suitable for detecting internal or latent damage in fruit [14]. Near-infrared hyperspectral imaging identifies changes in a sample’s internal structure and composition through differences in spectral response, providing an effective means of detecting low-level variations [15].

**Figure 2:**
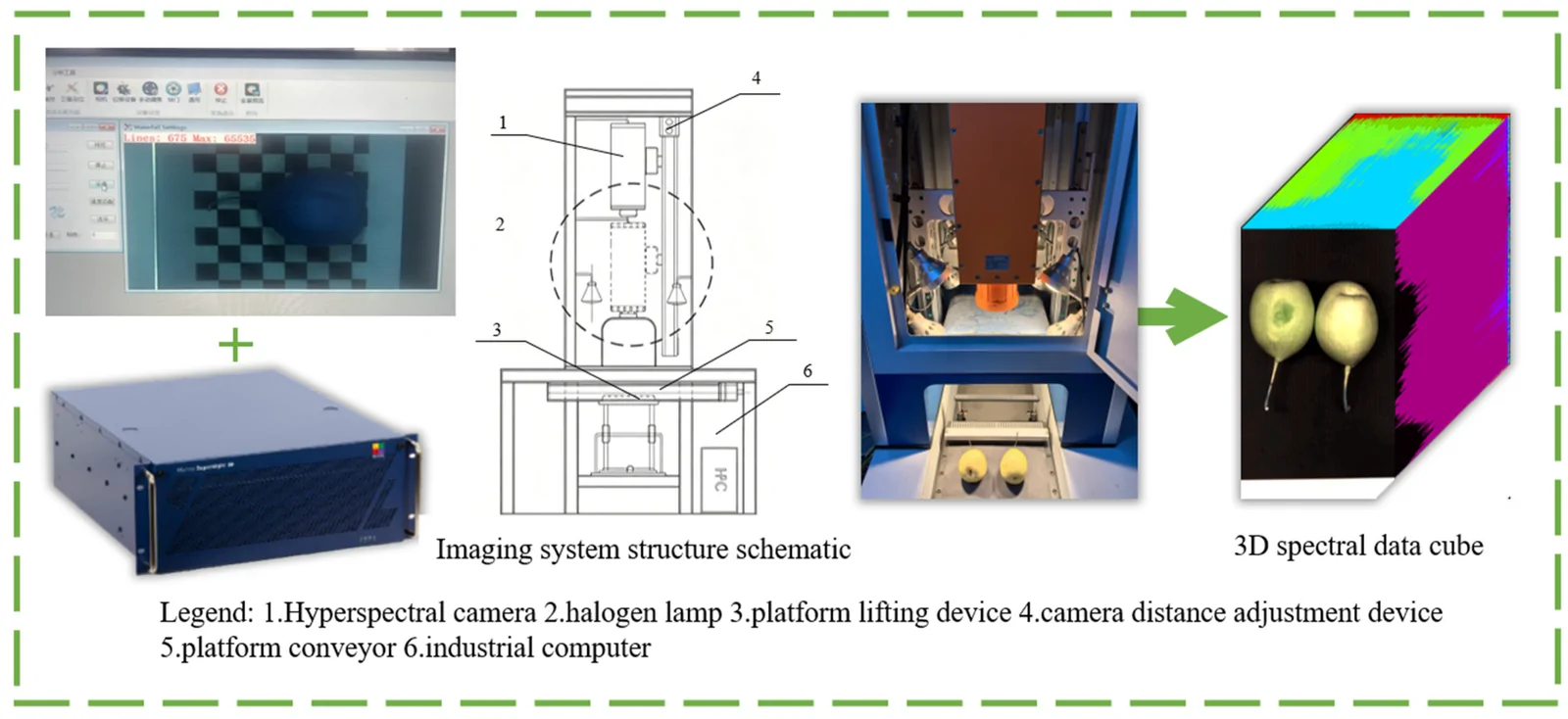
Schematic diagram of a hyperspectral imaging system.

**Figure 3:**
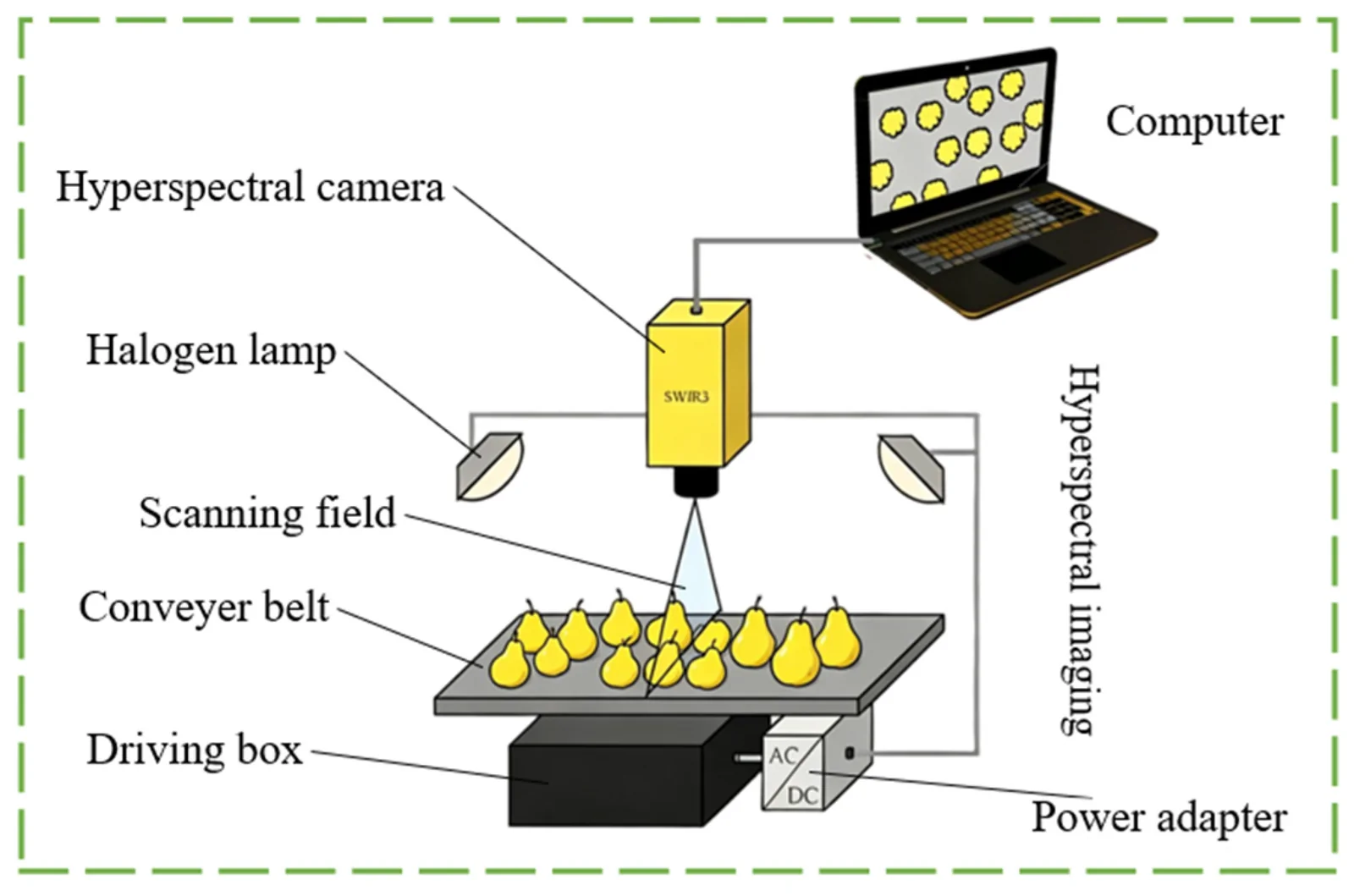
Schematic diagram of a line-scan hyperspectral image acquisition system.

This study utilised a line-scan short-wave infrared hyperspectral imaging system (1000–2500 nm), operated by a computer system and comprising the following main components: halogen lamps, a spectral camera, a platform lifting mechanism, a platform conveyor belt and a camera distance adjustment mechanism. The hyperspectral imaging system is shown in Figure 2. The spectrometer utilises a short-wave infrared camera and four halogen lamp light sources, arranged symmetrically at 45° angles, and employs an electric translation stage (speed 0.8 cm/s) to ensure the accuracy of line-scan image stitching. Its operating principle is illustrated in Figure 3: the camera captures a single row of the sample surface in a single acquisition, simultaneously recording the spectral response of each pixel in that row within the 1000–2500 nm range; whilst the sample stage moves horizontally at a constant speed, the camera scans row by row and the software stitches the data together to generate a three-dimensional hyperspectral data cube. The data cube obtained in this study comprises two spatial dimensions (x, y) and one spectral dimension (λ), with a spatial resolution of 384 × 600 pixels and a total of 288 spectral bands. Subsequent data analysis, dimensionality reduction and the construction of a lightweight CNN model were carried out using Python 3.9.16, along with NumPy, SciPy, scikit-learn and TensorFlow 2.12.

### 2.3. Selection and Preparation of Test Samples

This study selected the ‘Yali pear’ (Pyrus bretschneideri cv. Yali, hereinafter referred to as the Yali pear) as the test sample. This variety is one of the main pear cultivars widely grown in northern China and is prone to mechanical damage during storage and transport. The samples were harvested in September 2025 from an orchard in Shandong Province (36.48° N, 115.44° E). A total of 200 Yali pears were selected for the experiment; these were of similar size, with a single-fruit weight of 150–200 g, free from natural damage and with uniform skin colour. Following further selection, 180 of these were chosen as the experimental samples. From the selection of Yali pears with no natural damage, two typical types of damage were simulated: Mechanical scratch and Compression damage. The samples were divided into 60 undamaged fruits and 120 damaged fruits, comprising 60 with Mechanical scratch and 60 with Compression damage. Prior to hyperspectral imaging, all samples were washed using a specialised fruit and vegetable cleanser and then left in a well-ventilated area for 24 h to prevent moisture from affecting the spectral characteristics of the regions of interest (ROIs). To simulate Mechanical scratch, shallow wounds (3–5 cm in length and 0.5–1 mm in depth) were created randomly on the fruit surface using a blade; one wound was created per fruit. Compression damage was simulated by colliding two pears (resulting in cell rupture but no breakage of the epidermis), with one damage site created per fruit. Additionally, 300 Yali pears (150 healthy fruits and 150 damaged fruits) were procured from the same orchard during the same period; the damage preparation method was consistent with that of the training samples, and these were specifically designated for independent validation at the fruit level.

### 2.4. Black-and-White Correction

**Figure 4:**
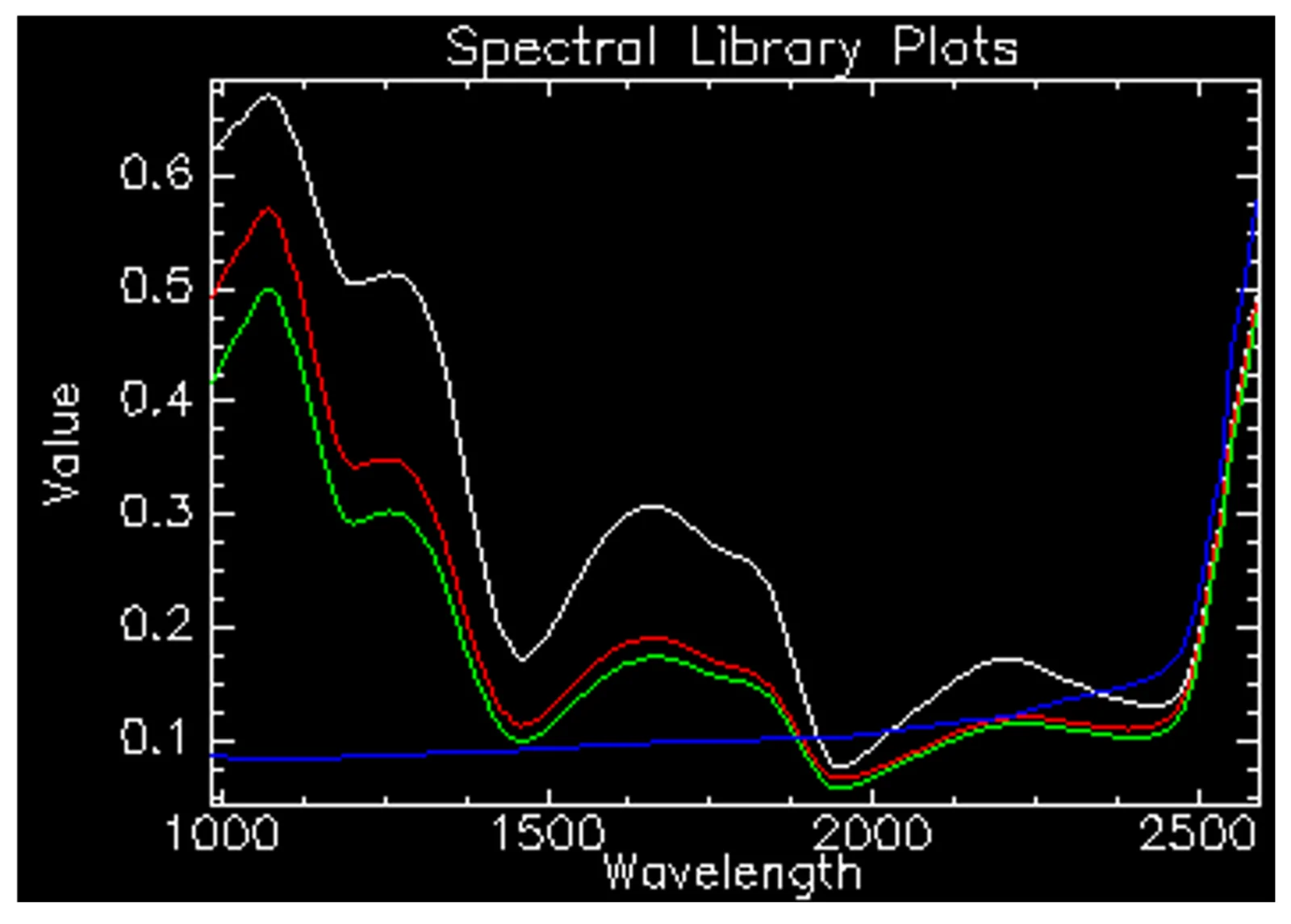
Calibrated sample reflectance curve.

To address issues such as sensor noise, uneven illumination and instrument drift, black-and-white calibration is employed, with the calculation method shown in Equation (1). Here, *Ic* denotes the calibrated image, *Ir* denotes the raw acquired image, *Iw* denotes the acquired whiteboard image, and *Id* denotes the acquired background board image. The core objective is to restore the true spectral reflectance or radiance of the object by calibrating the dark current (black field) and saturation signal (white field) of the image. Figure 4 shows the reflectance curve of a sample after correction.

(1) $$\mathrm{Ic}=\frac{\mathrm{Ir}-\mathrm{Id}}{\mathrm{Iw}-\mathrm{Id}}$$

### 2.5. ROI Annotation Rules and Sample Sizes for Each Category

**Figure 5:**
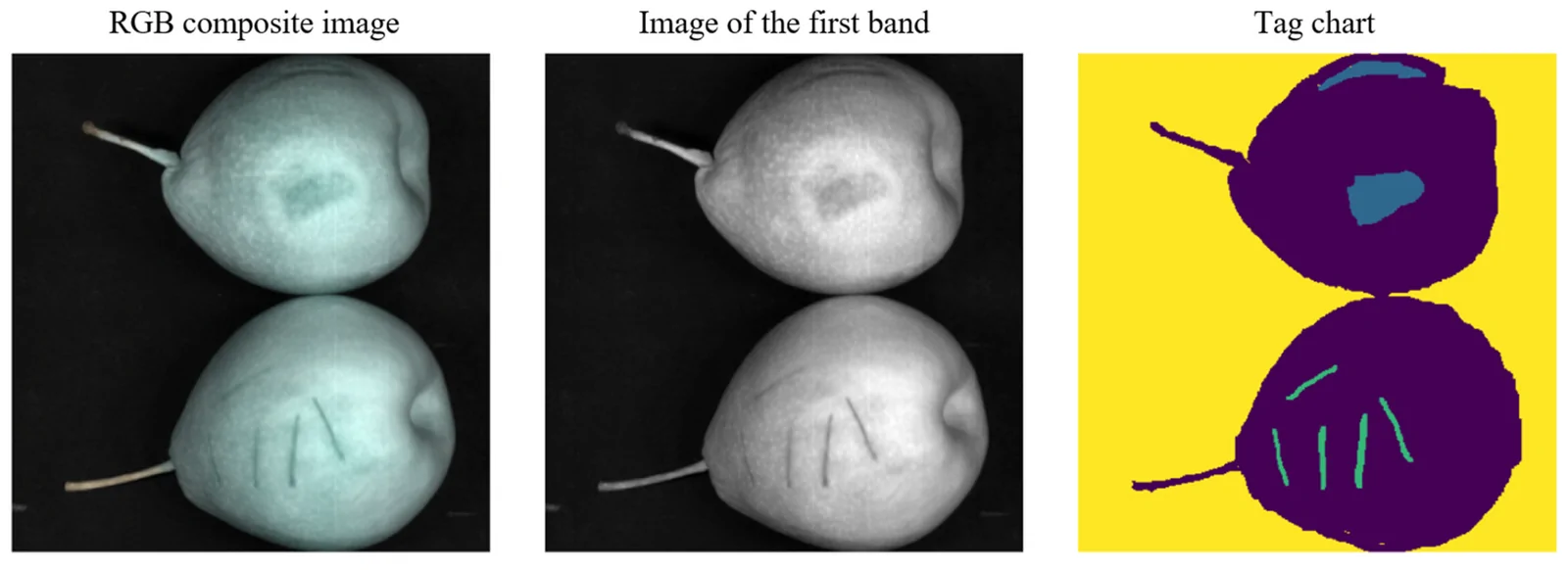
Examples of ROI annotations for the four sample categories.

ROI annotations were manually drawn using ENVI Classic 5.3 software by a trained researcher and reviewed region by region by a second researcher to ensure accurate boundaries and correct classifications. A conservative approach was adopted for the transition boundaries between healthy tissue and the lesion area: the healthy tissue ROI was selected as the area at least 2 mm from the edge of the lesion, whilst the lesion ROI covered only the core lesion area; pixels in the transition zone were not assigned to any category, in order to eliminate interference from samples with blurred boundaries on the classification model. The authenticity of the compression damage was confirmed by subepidermal browning observed 30 min after injury preparation; the browning area corresponded to the annotated ROI, eliminating the need for destructive validation. Figure 5 shows examples of ROI annotations for the four sample categories.

See Table 1 for details of the ROI annotation rules and sample sizes for each category. During the ROI annotation phase, the spatial heterogeneity and differences in area distribution across the fruit surface for each category were fully taken into account, and a differentiated annotation strategy was devised to ensure the representativeness and balance of the samples. The ‘intact tissue’ category had the widest range of sample sources; three regions were annotated per healthy fruit, whilst two healthy regions distant from the damaged areas were annotated per damaged fruit, totalling 420 ROIs. Each ROI covered an average of approximately 156 pixels, enabling the spectral variability across different parts of the fruit surface to be fully captured. For both Compression damage and Mechanical scratch, annotation focused on the core damaged areas, with one ROI annotated per fruit for each category, totalling 60 ROIs. The average number of pixels per ROI was 43.5 and 19.8 respectively; this difference in area reflects the actual morphological characteristics of the damage, with Compression damage exhibiting a surface-like spread whilst Mechanical scratch display a linear distribution. For the background category, one ROI was selected from each of the four corner regions of every hyperspectral image, totalling 720 ROIs, with an average of approximately 117 pixels per ROI, covering background spectral information from different spatial locations. Statistical analysis revealed that the total number of spectral data points extracted from all ROIs was 153,597. In accordance with the fruit-level independent partitioning scheme described in Section 2.6, all fruits were first divided into training, validation and test sets, after which spectral data was extracted from the fruits in each set. Ultimately, the training set contained 86,014 entries (56 per cent), the validation set contained 21,503 entries (14 per cent) and the test set contained 46,080 entries (30 per cent). The total number of pixels in the test set corresponds exactly to the total number of test samples reported in the subsequent confusion matrix, ensuring consistency and traceability in data statistics. This partitioning method ensures that all ROIs annotated on a single fruit are assigned to a single set, thereby guaranteeing that the validation and test results are not affected by pixel correlations within the fruit.

### 2.6. ROI: Fruit-Level Independent Data Segmentation Solution

Data partitioning treats each fruit as an independent unit; all pixel image blocks from a single fruit are assigned to a single set, ensuring that the training and test sets are completely independent at the level of biological samples [16,17]. Data partitioning is carried out prior to ROI annotation and pixel extraction; that is, the sequence of fruit segmentation → ROI annotation → pixel extraction is strictly followed, with the individual fruit serving as the smallest unit of segmentation. The 180 modelling fruits are divided into three subsets: a training set, a validation set and a test set. The key principle is that partitioning precedes pixel extraction; subsequently, ROI annotation and pixel sample extraction are carried out only within the fruit images of the corresponding subsets. It is strictly prohibited for pixels from the same fruit to appear across different subsets, thereby preventing data leakage caused by strong correlations between pixels from the same fruit. The specific partitioning ratios and fruit composition are shown in Table 2 below.

At the fruit level, the 180 Yali pear samples were divided into a training set (101, 56 per cent), a validation set (25, 14 per cent) and a test set (54, 30 per cent). The distribution of fruit categories across the sets remained broadly balanced, with 18 fruits of each category in the test set, ensuring that the evaluation process was not affected by category bias. Based on the ROI annotation rules in Table 1 and the aforementioned fruit-level division scheme, the number of ROIs for each category and their spectral distribution across the data sets are shown in Table 3. The training set comprises a total of 707 ROIs (86,014 pixel spectra), the validation set 175 ROIs (21,503), and the test set 378 ROIs (46,080), totalling 1260 ROIs (153,597), which is consistent with the global statistics in Table 1. Among these, the ‘intact tissue’ and ‘background’ categories comprise the largest numbers of pixels, with 65,520 and 84,280 respectively, providing the model with ample samples for learning spectral features; the ‘Compression damage’ and ‘Mechanical scratch’ categories contain relatively few damaged pixels, with 2610 and 1187 respectively, which corresponds to the actual situation where damaged areas account for a small proportion of the fruit’s surface. All ROIs annotated on a single fruit belong to a single set; this classification ensures that the pixels used for training and evaluation originate from entirely different individual fruits, thereby fundamentally avoiding the risk of performance overestimation caused by pixel correlation within a single fruit. It should be specifically noted that the independent biological replication unit in this study is the fruit. The pixel-level analysis utilised 153,597 pixel spectra extracted from 180 modelling fruits; however, the pixels do not constitute entirely independent biological samples—pixels within the same fruit share similar physiological states and spectral characteristics. Consequently, in our statistical presentation, we strictly distinguish between the ‘number of pixel-level observations’ (n = 153,597) and the ‘number of independent biological samples’ (n = 180 fruits). The fruit-level validation section utilised 300 completely independent fruits as independent biological samples.

### 2.7. Spectral Pre-Processing and Feature Extraction

The hyperspectral data collected for Yali pears in this study covers the 1000–2500 nm wavelength range, comprising a total of 288 spectral bands. This high-dimensional data suffers from issues such as spectral redundancy, noise interference and high computational complexity; using it directly for model training would reduce computational efficiency and affect classification stability [18]. To eliminate severe multicollinearity between spectral bands, this study first employed principal component analysis (PCA) to pre-process the raw spectral data, compressing the 288-dimensional spectrum into principal components that are mutually orthogonal and uncorrelated. This preserves the vast majority of spectral information whilst providing a unified and comparable input space for subsequent analysis. The PCA transformation matrix was fitted using only the training set data; specific implementation details are provided in Section 2.9.

Principal Component Analysis (PCA) achieves unsupervised dimensionality reduction by identifying the orthogonal directions with the greatest variance; Independent Component Analysis (ICA), on the other hand, assumes that the signal sources are statistically independent and extracts independent components that do not follow a Gaussian distribution. The core steps of PCA include data standardisation (Equation (2)), calculation of the covariance matrix (Equation (3)), eigenvalue decomposition (Equation (4)), selection of the first k principal components (Equation (5)), and projection-based dimensionality reduction (Equation (6)). Neither of the above methods utilises the class label information of the samples. Given that damage identification is essentially a supervised classification task, this study further introduces Linear Discriminant Analysis (R-LDA), which has the advantage of its supervised nature. It can specifically enhance class discrimination information, significantly improving the performance of subsequent classification models, and is particularly well-suited to classification tasks such as damage detection [19,20,21]. The core steps of R-LDA include the within-class divergence matrix (Equation (7)), the between-class divergence matrix (Equation (8)) and the solution (Equation (9)). R-LDA identifies the optimal projection direction by maximising the ratio of the between-class divergence matrix to the within-class divergence matrix, thereby ensuring that different damage categories are separated as much as possible in the low-dimensional space and that samples of the same category are clustered as closely as possible [22].

(2) $$x_{\mathrm{ij}}^{\prime }=\frac{x_{\mathrm{ij}}-u_{j}}{\sigma_{j}}$$

(3) $$C=\frac{1}{n-1}X^{T}X$$

(4) $$CV_{i}={\;\lambda }_{i}V_{i}$$

(5) $$V_{k}=\left[V_{1},V_{2},\cdots ,V_{k}\right]$$

(6) $$Y=XV_{k}$$

(7) $$S_{W}=\sum_{i=1}^{C}\sum_{x\in C_{i}}(x-m_{i})(x-m_{i}{)}^{T}$$

(8) $$S_{B}=\sum_{i=1}^{C}n_{i}(m_{i}-m)(m_{i}-m{)}^{T}$$

(9) $$S_{B}\omega =\lambda S_{W}\omega$$

### 2.8. Performance Comparison of Feature Extraction Strategies

**Figure 6:**
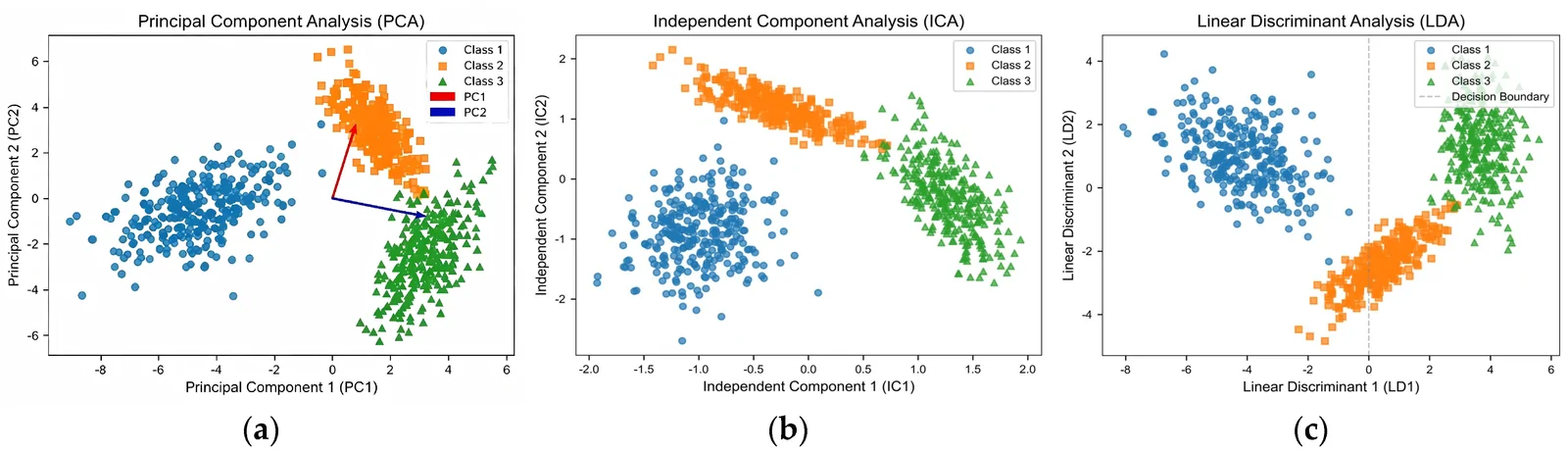
Schematic diagram of dimensionality reduction theory: (**a**) PCA; (**b**) ICA; (**c**) R−LDA.

Near-infrared spectroscopy combined with chemometric methods has been widely applied in sample classification and the analysis of quality indicators, which also highlights the importance of discriminant feature extraction for spectral classification tasks [23]. A schematic diagram of dimensionality reduction is shown in Figure 6. PCA and ICA aim to maximise data variance and extract independent signals, respectively; although they achieve dimensionality reduction, their ability to distinguish between different damage categories is limited, with feature overlap persisting in some categories; in contrast, R-LDA aims to maximise inter-class distance and minimise intra-class variance, projecting samples into a lower-dimensional feature space with greater discriminative power, thereby achieving clear separation of different damage categories and demonstrating superior class discrimination performance.

**Figure 7:**
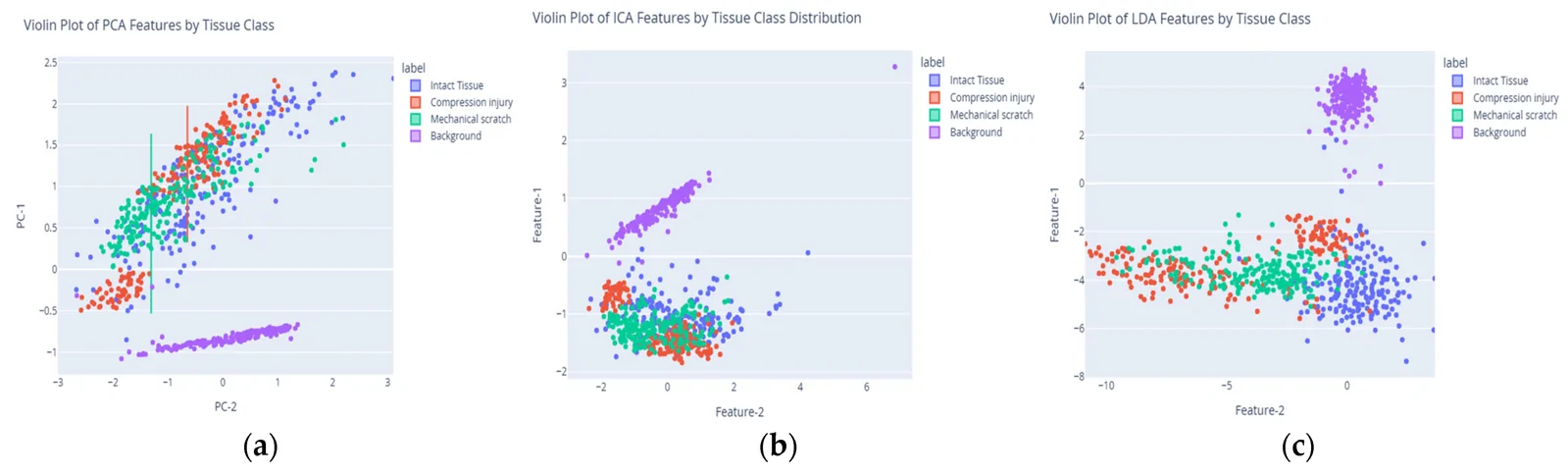
Scatter plot of feature distributions: (**a**) PCA; (**b**) ICA; (**c**) R-LDA.

A scatter plot of the actual feature distribution is shown in Figure 7, whilst a comparison of the feature separation performance of different feature extraction strategies is presented in Table 4. Analysis of the above results reveals that R-LDA achieved the highest Fisher’s discrimination criterion (J = 5.0275) and separation index (SI = 2.3543), indicating that its projected feature space exhibits the greatest inter-class distance and the lowest intra-class dispersion. PCA ranks second (J = 2.8694, SI = 2.0064), whilst ICA performs the weakest in this task (J = 0.4326, SI = 1.3530). This may be attributed to the fact that ICA assumes the source signals are statistically independent, whereas adjacent bands in hyperspectral data exhibit strong continuous correlations; this assumption may not hold strictly for this dataset, resulting in the projected directions failing to effectively distinguish between damage categories. In terms of computation time, all three strategies can be completed within 4 s, meeting the requirements of an offline training scenario.

### 2.9. Experimental Setup and Notes on Reproducibility

This study employs Regularised Linear Discriminant Analysis (R-LDA), implemented using the LinearDiscriminantAnalysis class from the scikit-learn library, with the solver set to ‘SVD’. Standard LDA determines the optimal projection direction by maximising the ratio of inter-class divergence to intra-class divergence, as detailed in Equation (10). Here, *S_b_* denotes the inter-class divergence matrix and *S_w_* denotes the intra-class divergence matrix. Under high-dimensional, small-sample conditions, when LDA is applied directly to raw spectra, *S_w_* may become singular and its inverse cannot be computed. Standard LDA determines the optimal projection direction by maximising the ratio of inter-class divergence to intra-class divergence. Under high-dimensional, small-sample conditions, when LDA is applied directly to raw spectra, the intra-class divergence matrix *S_w_* may become singular and its inverse cannot be computed. The R-LDA adopted in this study resolves this issue through a dual regularisation mechanism. The first regularisation step involves PCA truncation pre-processing: after the raw spectra undergo PCA transformation, the feature dimension is reduced from 288 to 8, which is far smaller than the number of training samples, thereby ensuring that *S_w_* is full-rank and invertible. The second regularisation is solved via truncated SVD: when the solver is set to ‘svd’ in scikit-learn’s LinearDiscriminantAnalysis, a truncated singular value decomposition is performed on *S_w_*, retaining only singular values greater than the threshold τ, thereby approximating *S_w_* as a low-rank matrix. This low-rank approximation is mathematically equivalent to spectral regularisation—which suppresses the noise amplification effect in small singular value directions by constraining the rank of the matrix—and is similar in mathematical effect to ridge regularisation. The automatic discarding of singular values close to zero further enhances the numerical robustness of the solution. Compared with traditional LDA, R-LDA is solved in the truncated feature space obtained via PCA, thereby avoiding the instability associated with directly inverting high-dimensional collinear spectra; compared with PCA-LDA, R-LDA introduces an additional SVD truncation step during the LDA phase, rather than simply applying standard LDA after PCA dimensionality reduction.

(10) $$J(\omega )=\frac{\omega^{T}S_{b}\omega }{\omega^{T}S_{\omega }\omega }$$

This study employs Regularised Linear Discriminant Analysis (R-LDA), implemented using the LinearDiscriminantAnalysis module from the scikit-learn library, with the solver set to ‘SVD’. The numerical stability of R-LDA is ensured by a dual mechanism. Firstly, the PCA truncation pre-processing reduces the original 288-dimensional spectra to 8 orthogonal and uncorrelated principal components, ensuring that the feature dimension is far smaller than the number of training samples, thereby allowing the intraclass covariance matrix to be inversely calculated stably; Secondly, the SVD solver performs truncated singular value decomposition on the within-class covariance matrix, further enhancing the numerical robustness of the solution. The term ‘R-LDA’ is used to emphasise the central role of the truncated PCA pre-processing step within the overall regularisation strategy. The R-LDA projection matrix is fitted using only the training set data.

The PCA transformation matrix is fitted strictly using only the training set data (pca.fit(X_train)), whilst the validation and test sets are merely mapped forward using the learned transformation matrix (pca.transform(X_test)) and are not involved in parameter estimation. The number of principal components retained is determined based on a cumulative variance contribution of ≥99%. Based on the fitting results from the training set, the cumulative variance explained by the first eight principal components reached 99.3%; therefore, eight principal components were retained for all subsequent analyses. Preliminary validation showed that when k was increased to 10 or 12, the subsequent classification performance improved by less than 0.2%, whilst reducing k to 5 or 6 resulted in a performance decline of approximately 1–2%, indicating that 8 dimensions strike a reasonable balance between information retention and computational efficiency. As the training set was fixed via deterministic allocation during the data preparation stage, and PCA is a deterministic algorithm, the PCA pre-processing remained entirely consistent across all five runs with different random seeds.

PCA pre-processing (fitted using the training set only) provides a unified orthogonal feature space for all methods. This study compared three feature extraction strategies: PCA-CNN, which directly selects the top eight principal components to construct spectral image patches as input to a lightweight CNN and serves as the baseline strategy to assess the upper limit of performance achievable through direct classification following unsupervised dimensionality reduction, and ICA-CNN, which employs Independent Component Analysis (ICA) to extract statistically independent features, with the number of independent components set to 8. Three settings—5, 8 and 12 dimensions—were examined on the training set, yielding pixel-level classification accuracies of 94.32%, 95.10% and 94.78% respectively. As 8 dimensions achieved the best performance, this was selected as the final parameter for ICA. ICA dimension testing was conducted on the training set using 5-fold cross-validation, with PCA-CNN serving as the classifier to evaluate performance at different dimensions, ensuring that the testing process did not involve information from the validation or test sets. The ICA model maintains the same input dimension as PCA-CNN to ensure a fair comparison; R-LDA-CNN utilises R-LDA to extract discriminative features, outputting three discriminant components (number of classes minus one), and constructs a lightweight CNN using spectral image patches as input. The dimensionality changes throughout the complete dimension reduction process are as follows: 288 dimensions in the original spectrum → 8 dimensions after PCA pre-processing → 8 dimensions after ICA feature extraction/3 dimensions after R-LDA feature extraction.

Fruit-level sorting employs a voting mechanism based on the proportion of damaged pixels per fruit to map pixel-level prediction results to fruit-level labels. The proportion of damaged pixels is defined as the sum of the number of pixels affected by Compression damage and Mechanical scratch, divided by the total number of pixels in the fruit area (excluding background pixels); when this proportion exceeds the threshold *θ*, the fruit is classified as damaged. In this study, *θ* = 5 per cent; all three models (PCA-CNN, ICA-CNN and R-LDA-CNN) employ the same threshold to ensure a fair comparison. This threshold was determined by analysing the fruits in the training set to calculate the distribution of the proportion of actual damaged pixels relative to the total number of pixels in each fruit, and selecting a lower quantile from this distribution as the threshold. This ensures that the vast majority of damaged fruits are correctly labelled during the training phase. This threshold was determined prior to the start of the experiment, and no fine-tuning or selection was carried out on the validation or test sets, thereby avoiding any bias in the fruit-level evaluation results caused by information leakage from the test set.

### 2.10. Building a Convolutional Neural Network Model

**Figure 8:**
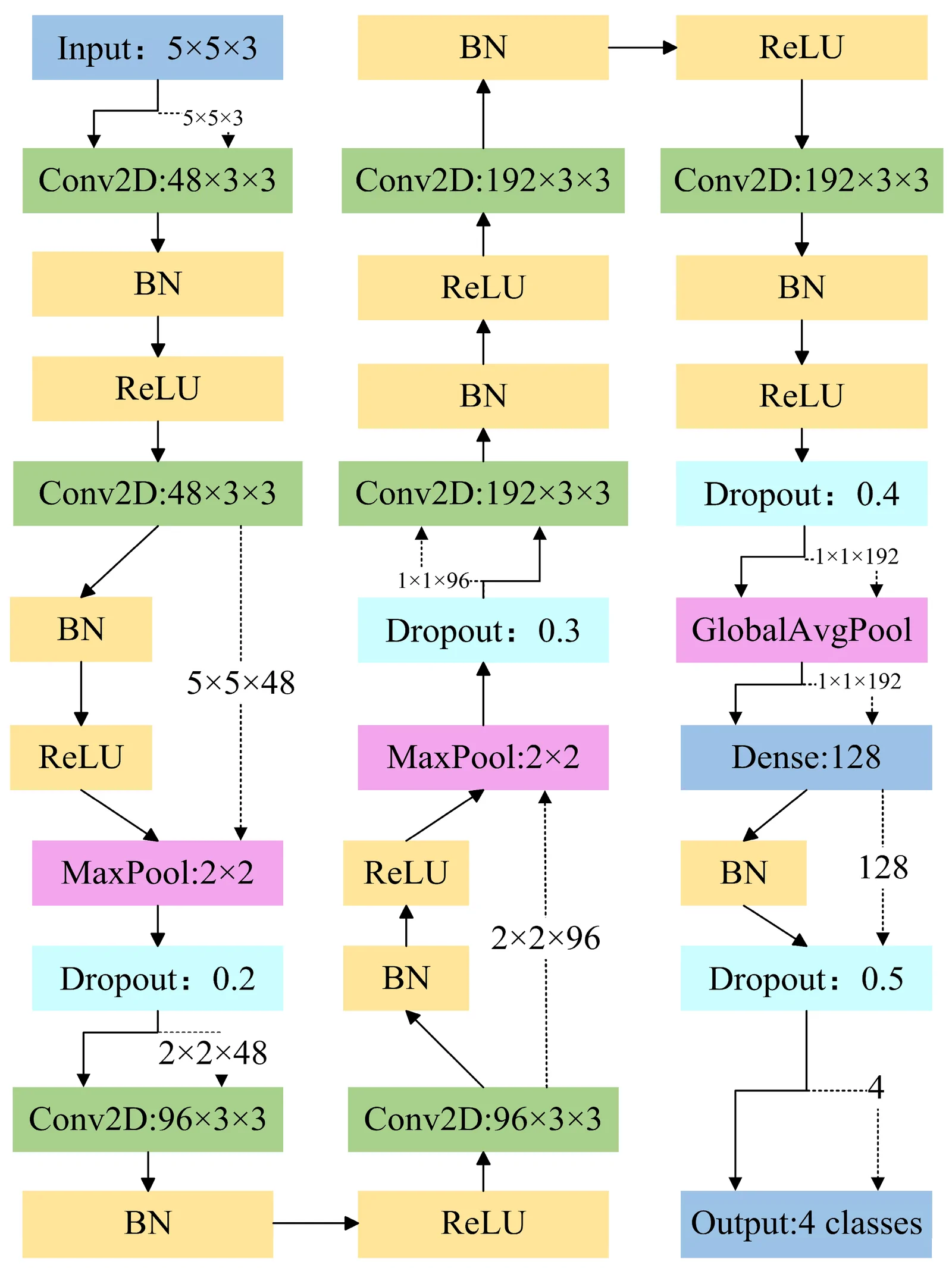
Overall architecture diagram of a lightweight convolutional neural network.

To address the three challenges in the hyperspectral damage classification of Yali pears—namely, weak damage features, high real-time performance requirements, and class imbalance leading to overfitting—this paper designs a lightweight convolutional neural network, the overall architecture of which is shown in Figure 8. The three feature extraction strategies share an identical CNN architecture, with only the number of input channels in the first layer adapted according to the feature dimensions: the inputs for the PCA-CNN and ICA-CNN are 5 × 5 × 8 spatio-spectral image patches (8 principal components or independent components), whilst the input for the R-LDA-CNN is a 5 × 5 × 3 spatio-spectral image patch (3 discriminant components). The output consists of prediction probability vectors for four classes: ‘intact tissue’, ‘Compression damage’, ‘Mechanical scratch’ and ‘background’. The model employs a hierarchical feature extraction strategy, in which three convolutional blocks progressively extract features from local texture to high-level semantic features, and the classification results are output via global average pooling and a lightweight classification module. To suppress overfitting whilst maintaining a lightweight model, L2 weight regularisation (*λ* = 1 × 10^−4^) is applied to each convolutional layer. Additionally, dropout with a probability ranging from 0.2 to 0.5 is introduced between convolutional blocks and fully connected layers, supplemented by batch normalisation to accelerate convergence and stabilise training. During training, a label smoothing strategy with *ε* = 0.1 is employed to further mitigate the risk of overfitting caused by class imbalance. Detailed parameter configurations for each module are shown in Table 5.

The model was built using the TensorFlow 2.12 framework (Python 3.9.16), on a computing platform configured with an Intel Core i7-13700K CPU, 32 GB of RAM and an NVIDIA GeForce RTX 4070 Ti GPU (12 GB VRAM). Training utilised the Adam optimiser, with an initial learning rate of 0.001 and a batch size of 64; an adaptive learning rate decay strategy was employed (the learning rate is halved when the validation loss fails to decrease for five consecutive epochs), and an early stopping mechanism was set to terminate training and revert to the optimal weights if the validation loss shows no improvement for 15 consecutive epochs; the global random seed was fixed at 42 to ensure reproducibility. The model comprises approximately 1.0 × 10^6^ trainable parameters, with a storage size of approximately 3.8 MB (FP32). On the same platform, a complete inference for a single hyperspectral image (384 × 600 pixels, 288 bands) takes approximately 1.0 s, encompassing the entire process including PCA pre-processing, R-LDA projection, neighbourhood block construction and CNN forward propagation. The aforementioned lightweight parameter scale and sub-second inference speed demonstrate potential for near-real-time applications. It should be noted that actual industrial sorting performance also depends on factors such as image acquisition time, conveyor belt speed, fruit spacing and system-level processing workflows; the inference time for a single image is merely one aspect of this process. Through further engineering optimisations, such as model quantisation and TensorRT acceleration, inference time is expected to be reduced to the millisecond range, thereby better meeting the demands of high-throughput sorting production lines.

### 2.11. A Comparative Analysis of CNN Performance Versus Traditional Classifiers

To further verify whether the performance advantage of lightweight CNNs on R-LDA-dimension-reduced features stems from their network architecture itself, rather than relying solely on the discriminative power of the R-LDA features, this study conducted additional pixel-level classification comparison experiments using three traditional classifiers—Support Vector Machines (SVM, RBF kernel), Random Forest (n_estimators = 100) and Partial Least Squares Discriminant Analysis (PLS-DA). The results are shown in Table 6.

As shown in Table 6, when fed the same 3-dimensional LDA discriminant features, the classification accuracy of the lightweight CNN (97.98 per cent) was higher than that of SVM (91.25 per cent), PLS-DA (90.52 per cent) and Random Forest (89.80 per cent), with a performance improvement of 6.5–8.2 percentage points. The performance of the three traditional classifiers is relatively close and significantly lower than that of the CNN, indicating that the upper limit of accuracy achievable through a pixel-by-pixel independent classification strategy, relying solely on the linear separability of R-LDA features, is limited. The performance advantage of the lightweight CNN stems primarily from its ability to utilise spatial contextual information. SVM, Random Forest and PLS-DA take flattened 3-dimensional feature vectors as input; essentially, they classify each pixel independently, ignoring the spatial correlations between a pixel and its neighbourhood. In contrast, CNNs take 5 × 5 × 3 spatial–spectral image patches as input, and the convolution operation can simultaneously encode the spectral features of the central pixel and the spatial distribution patterns of its surrounding neighbourhood pixels. For the task of identifying mechanical damage in Yali pears, there is a gradual transition zone between areas of compression damage and intact tissue, whilst mechanical scratch exhibit distinct linear directional characteristics. This spatial structural information is entirely lost within a framework of independent pixel-by-pixel classification; however, CNNs effectively capture such combined spatial–spectral features through the local receptive fields of convolutional kernels and their hierarchical feature extraction capabilities, thereby achieving a significant leap in classification accuracy. The above comparative results further validate, from a methodological perspective, the rationale for adopting a lightweight CNN rather than a traditional classifier in this study. R-LDA dimensionality reduction provides a high-quality discriminative feature space, whilst the CNN, building upon this, further unlocks the potential of these features through its ability to model spatial context; the synergistic interaction between the two constitutes the technical foundation for the performance advantages of this method.

## 3. Results and Discussion

In this study, PCA was used beforehand to eliminate multicollinearity among the spectral variables, thereby providing R-LDA with orthogonal and uncorrelated input features, ensuring a fair comparison of the three feature extraction strategies under consistent conditions. The projection matrices for all three methods were trained exclusively using the spectral data from the training set, comprising a total of 86,014 spectra (36,691 from intact tissue, 1462 from Compression damage, 665 from Mechanical scratch, and 47,196 from the background).

### 3.1. A Comparison of Pixel-Level Classification Performance Using Different Feature Extraction Strategies Combined with a Lightweight CNN

To assess the sensitivity of the model’s performance to random initialisation and batch shuffling, this paper employs a strategy of independent repeated training with multiple random seeds. Based on a fixed training/test set split, the three feature extraction strategies combined with the lightweight CNN model were trained and tested independently using five different random seeds (42, 123, 456, 789, 1024). The hyperparameter configurations were identical for each training run, and the pixel-level classification performance is reported as the mean of five replicate experiments along with a 95% confidence interval. Given the limited number of repetitions (n = 5), the confidence intervals were calculated using the t-distribution (95% CI = mean ± 2.776 × standard error) to more conservatively reflect the uncertainty of estimates under small sample sizes. This method objectively reflects the central tendency and stability of model performance, avoiding conclusions based on the randomness of a single experiment, whilst conducting comparative experiments under strictly controlled conditions regarding the division of training and test sets and the number of training iterations. The pixel-level classification performance reported in this section is based on 86,014 training pixel spectra, 21,503 validation pixel spectra and 46,080 test pixel spectra. These pixels were sourced from 180 independent fruits, comprising 101 in the training set, 25 in the validation set and 54 in the test set. Pixel-level metrics reflect the model’s discriminative ability at the level of individual pixels, the independence of which is ensured by the fruit-level partitioning protocol.

The pixel-level classification performance of the three feature extraction strategies—lightweight CNN models—on the test set is shown in Table 7. The R-LDA-lightweight CNN model achieved the best classification accuracy, with a mean of 97.98% across five replicate experiments and a 95% confidence interval of [97.30%, 98.66%], which is higher than the 95.70% (95% CI: 94.52–96.88%) achieved by the PCA model and the 95.10% (95% CI: 93.78–96.42%) achieved by the ICA model. In terms of convergence performance, the mean training loss of the R-LDA model was only 0.040, with a confidence interval of [0.030, 0.050], the narrowest among the three models. This indicates that its supervised discriminative features not only improved classification accuracy but also enhanced the stability and convergence consistency of model training. The ICA model had the widest confidence interval for accuracy (half-width 1.32%) and the greatest fluctuation in loss values (half-width 0.018), reflecting the relative lack of discriminative power in unsupervised feature extraction, which resulted in the model being more sensitive to random initialisation.

An analysis of training dynamics reveals significant differences in the impact of the three feature extraction strategies on the model’s convergence process. The PCA + lightweight CNN approach exhibited a low initial accuracy, a slow decline in validation loss, and severe fluctuations in the later stages. This stems from the fact that PCA maximises global variance; the retained principal components may not necessarily be relevant to the classification task and may instead introduce redundant information, thereby increasing training instability. Although the loss for ICA + Lightweight CNN tends to stabilise in the later stages, its overall classification accuracy is the lowest among the three, remaining at a ‘low and stable’ level. This indicates that the independent components extracted contain category-irrelevant noise, thereby weakening the model’s discriminative power. In contrast, R-LDA combined with a lightweight CNN demonstrated the optimal convergence characteristics throughout the training process: accuracy improved steadily, validation loss decreased rapidly and remained at an extremely low level, and performance fluctuations between batches were minimal. This is attributable to R-LDA, which aims to maximise inter-class divergence whilst minimising intra-class divergence. Through the synergistic effect of supervised learning and dual regularisation, it directly extracts the most discriminative low-dimensional features, effectively reducing the model’s learning difficulty and suppressing overfitting. In summary, the experimental results demonstrate that, leveraging the advantages of supervised learning, the discriminative features extracted by R-LDA outperform those of PCA and ICA in terms of both the Fisher criterion and the separation index. Furthermore, R-LDA outperforms PCA and ICA in terms of training stability and convergence efficiency. It is able to more effectively enhance inter-class differences and reduce intra-class dispersion, thereby providing more suitable feature inputs for the lightweight CNN and better suiting the task of distinguishing between the four types of tissue samples in this study.

### 3.2. Analysis of Classification Performance Based on the Confusion Matrix

**Figure 9:**
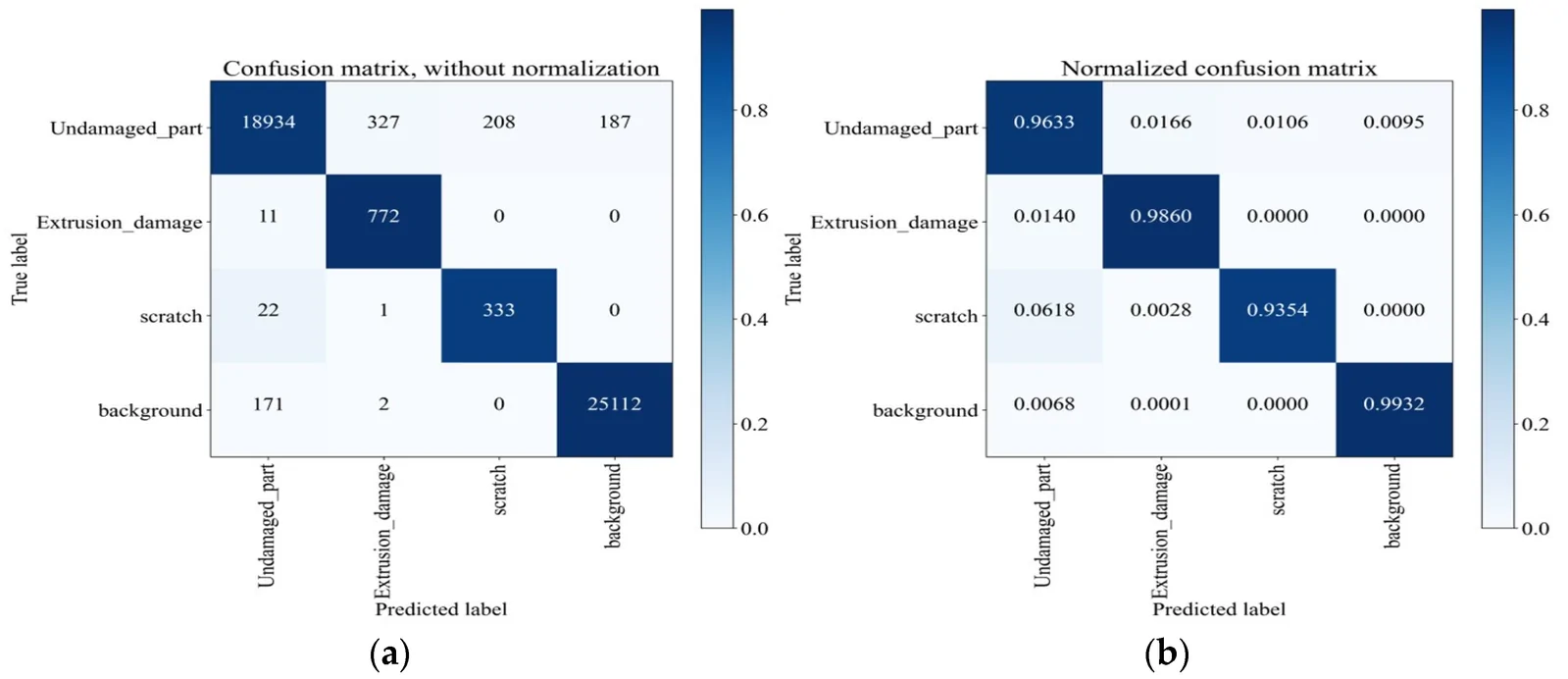
Original confusion matrix and normalised confusion matrix: (**a**) original confusion matrix; (**b**) normalised confusion matrix.

To further analyse the model’s recognition performance across different categories, this paper utilises confusion matrices to evaluate the classification results of the R-LDA + lightweight CNN model; the original confusion matrix and the normalised confusion matrix are shown in Figure 9, respectively. The values on the main diagonal of the normalised confusion matrix represent the recall rates for each category (i.e., the proportion of instances in each category that are correctly identified), with recall rates of 96.33 per cent, 98.60 per cent, 93.54 per cent and 99.32 per cent for intact tissue, Compression damage, Mechanical scratch and background, respectively. To further evaluate the classification performance for each category, Table 8 reports the precision, recall and F1 scores for each category achieved by R-LDA + lightweight CNN on the test set.

As can be seen from Table 8, the ‘intact tissue’ and ‘background’ classes exhibit high precision and recall rates, with F1 scores exceeding 97 per cent in both cases. The recall rates for ‘Compression damage’ and ‘Mechanical scratch’ reached 98.60 per cent and 93.54 per cent respectively, indicating that the model is highly effective at detecting damaged pixels; however, the precision rates for these two categories are relatively low (72.42 per cent and 56.35 per cent respectively). This is because damaged pixels account for an extremely small proportion of the test set; when a small number of intact tissue or background pixels are misclassified as damaged, the impact on precision is far greater than that on recall. After excluding the background class, the overall accuracy for the three classes (intact tissue, Compression damage and Mechanical scratch) was 96.37 per cent, with a Balanced Accuracy of 96.16 per cent, indicating that the model’s ability to distinguish damage in fruit tissue was not overestimated due to the high accuracy of the background class.

The classification results show that the model exhibits a high level of discrimination for background areas and areas of Compression damage, indicating that the discriminative features obtained after R-LDA dimensionality reduction effectively characterise the internal tissue changes caused by Compression damage. The recognition accuracy for the Mechanical scratch category is relatively low, with the primary source of error being the misclassification of some minor scratch as healthy tissue. This may be due to the spectral response of minor scratch areas being quite similar to that of the surrounding healthy epidermis, whilst the presence of transitional tissue zones at the edges of the scratch leads to some discrepancies between manual annotation and model predictions. Overall, misclassifications by the model are primarily concentrated between intact tissue and minor damage, whilst there is less confusion between Compression damage and Mechanical scratch, indicating that this method is effective at distinguishing between surface-level damage and internal Compression damage. Furthermore, the pixel-level classification task in this study exhibits a certain degree of class imbalance. As shown in Table 3 above, the number of pixels corresponding to Compression damage and Mechanical scratch is far fewer than those for intact tissue and background, consistent with the fact that damaged areas account for a relatively small proportion of the image. During training, label smoothing, L2 regularisation and dropout were employed to mitigate this effect; the recognition accuracies for the two damage categories reached 98.60 per cent and 93.54 per cent respectively, indicating that these measures were effective. However, with only 665 training pixels for Mechanical scratch, feature learning may not have been sufficiently thorough; this could be improved in future work by expanding the sample size.

**Figure 10:**
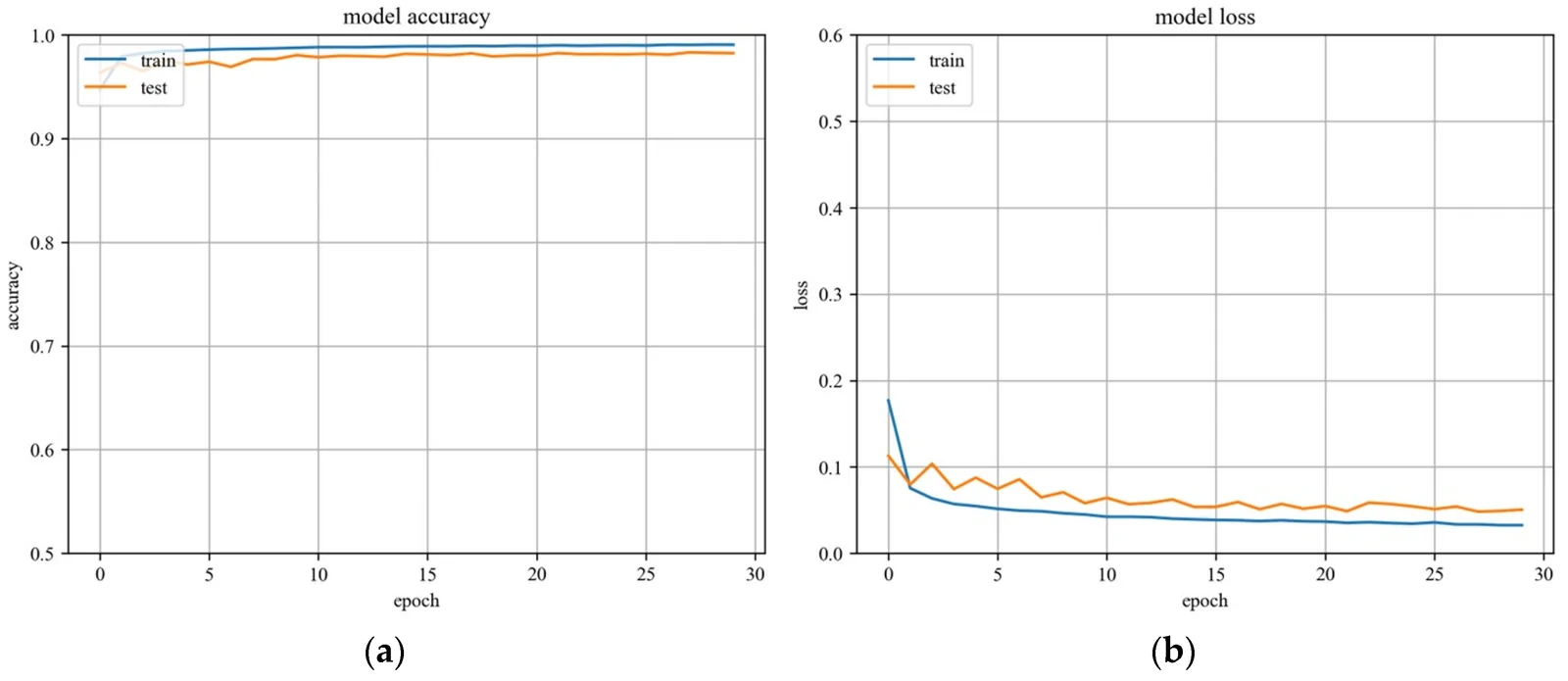
Model accuracy and model loss: (**a**) model accuracy; (**b**) model loss values.

The changes in accuracy and loss values during the training of the R-LDA + lightweight CNN model are shown in Figure 10. The PCA + lightweight CNN model exhibited a low accuracy rate during the early stages of training, with validation loss decreasing at a slow pace; slight fluctuations were observed in the later stages, indicating that the variance information extracted by PCA was not sufficiently targeted for damage class discrimination. The ICA + lightweight CNN model demonstrated relatively low overall classification performance, with validation loss tending to stabilise in the later stages of training, suggesting that the independent components extracted by ICA may contain noise information unrelated to the classes. In contrast, the R-LDA + lightweight CNN model exhibited a more stable training process, with both training and validation accuracies improving in tandem, whilst the loss value decreased rapidly and remained relatively consistent. This suggests that the discriminative features extracted by R-LDA effectively reduce the model’s learning difficulty and enhance its generalisation ability.

### 3.3. Visual Analysis of Classification Results

**Figure 11:**
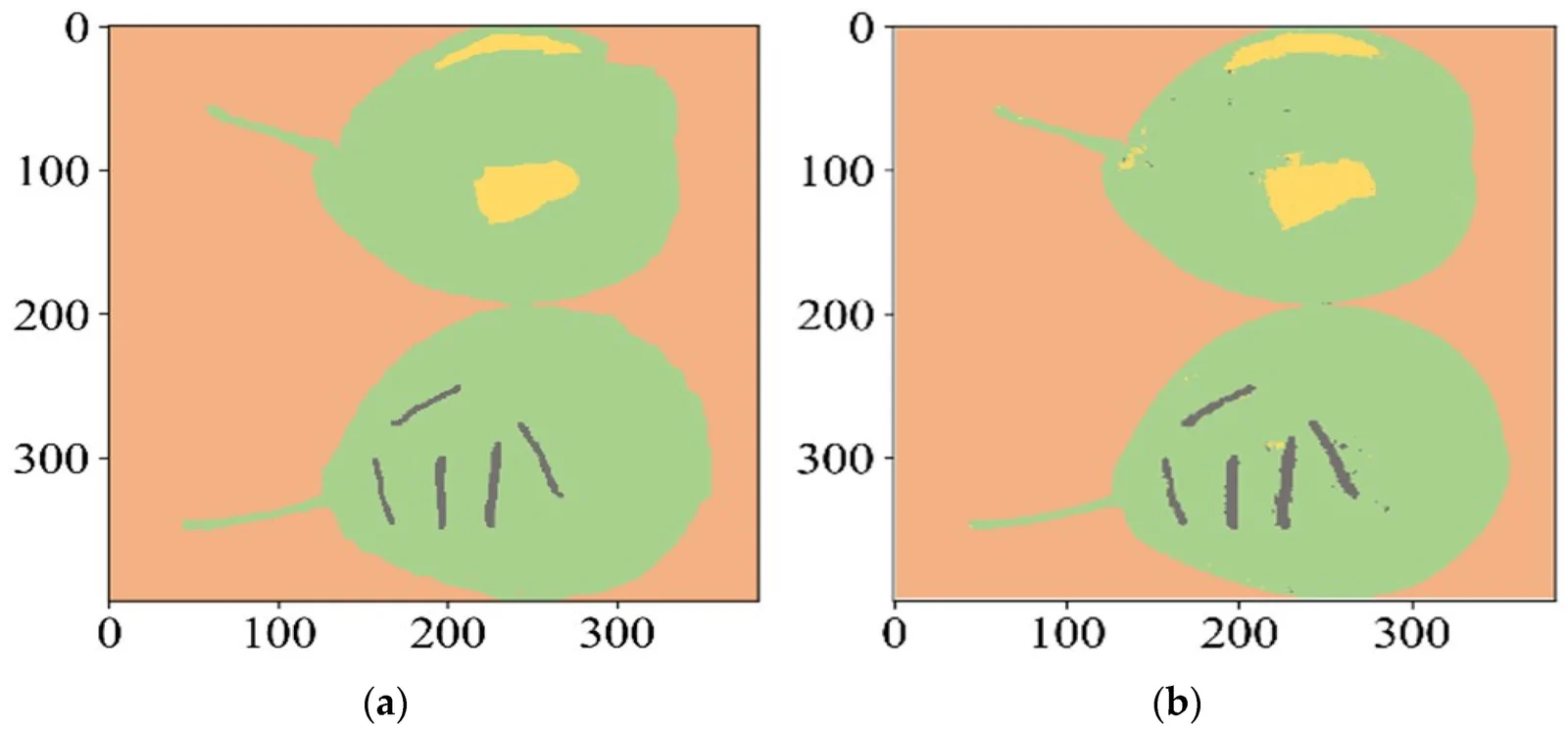
Manually labelled images and classification results: (**a**) manually labelled images; (**b**) rendering of the classification.

Figure 11 shows, respectively, the manually annotated results for damaged areas on Yali pears and the pixel-level classification predictions from the R-LDA + lightweight CNN model; the visual comparison between the two provides a direct basis for evaluating the model’s performance. In terms of spatial distribution consistency, the model’s predictions exhibit a high degree of spatial alignment with the manually annotated results, validating the effectiveness of this framework in the task of Yali pear damage detection. Specifically, the model demonstrates outstanding performance in recognising two typical types of damage: for mechanical scratch—which feature linear boundaries—the model is able to accurately capture gradient changes along the edges of the damage, generating linear responses that closely match the manual annotations. No obvious discontinuities, offsets or excessive expansion were observed, indicating that the model’s sensitivity to linear structures and its localisation accuracy have both reached an optimal level; for Compression damage—a typical area-based defect—the model achieved relatively comprehensive regional coverage; the predicted contours, geometric shapes and areas of the regions all maintained good consistency with the manually annotated results, effectively delineating the overall extent of the damage without any obvious oversegmentation or undersegmentation issues. The above results demonstrate that R-LDA discriminative feature extraction effectively retains the discriminative spectral features within the hyperspectral data, whilst the lightweight CNN fully exploits spatial neighbourhood information on this basis. The synergistic interaction between the two enables the model to maintain stable and accurate recognition performance when faced with damage types exhibiting different morphological characteristics. Overall, the model performed exceptionally well in terms of the accuracy of damage region localisation, the precision of boundary delineation, and its adaptability to different damage types, thereby validating the feasibility and effectiveness of this framework for the pixel-level recognition of damage in Yali pears.

However, closer examination of the image details reveals that the model still exhibits a small amount of prediction error, and these errors display distinct distribution characteristics: they are primarily concentrated at the edges of the lesions and in the transition zones between healthy and damaged tissue. This phenomenon is not coincidental; its causes can be explained in terms of both data characteristics and annotation bias. On the one hand, the spectral responses of adjacent tissues in hyperspectral images exhibit continuous transitions; the spectral characteristics of damaged and healthy regions are not sharply delineated but rather present gradual spectral gradients. This leads to the model being susceptible to spectral confusion during the classification of boundary pixels, making it difficult to establish an absolutely clear discriminatory boundary at the critical point between damaged and healthy tissue. On the other hand, during the manual annotation process, annotators’ delineation of damage boundaries is subject to a degree of subjectivity. Particularly in the early stages of damage or when the edges are visually indistinct, discrepancies in boundary localisation may arise not only between different annotators but also between different annotation batches by the same annotator. Such annotation bias introduces boundary noise, which in turn affects the model’s learning performance and ultimately manifests as classification errors in edge regions within the prediction results. Despite the aforementioned local errors, the R-LDA + lightweight CNN model demonstrated excellent overall performance in the Yali pear damage detection task. The model is not only capable of accurately identifying damage types with different morphological characteristics but also, whilst maintaining detection accuracy, significantly reduces model parameters and computational complexity through its lightweight convolutional design. By balancing computational efficiency with detection performance, it provides technical feasibility for practical online detection applications. Overall, the visualisation results fully validate the feasibility and reliability of this method in the non-destructive testing of damage to Yali pears. At the same time, they highlight that there is still room for optimisation in the model’s handling of boundary regions, providing a clear theoretical and experimental basis for future optimisation directions such as the introduction of edge enhancement strategies, multi-scale feature fusion, or active learning-based annotation correction.

### 3.4. Verification of the Generalisability of Independent Fruit-Level Sorting

**Figure 12:**
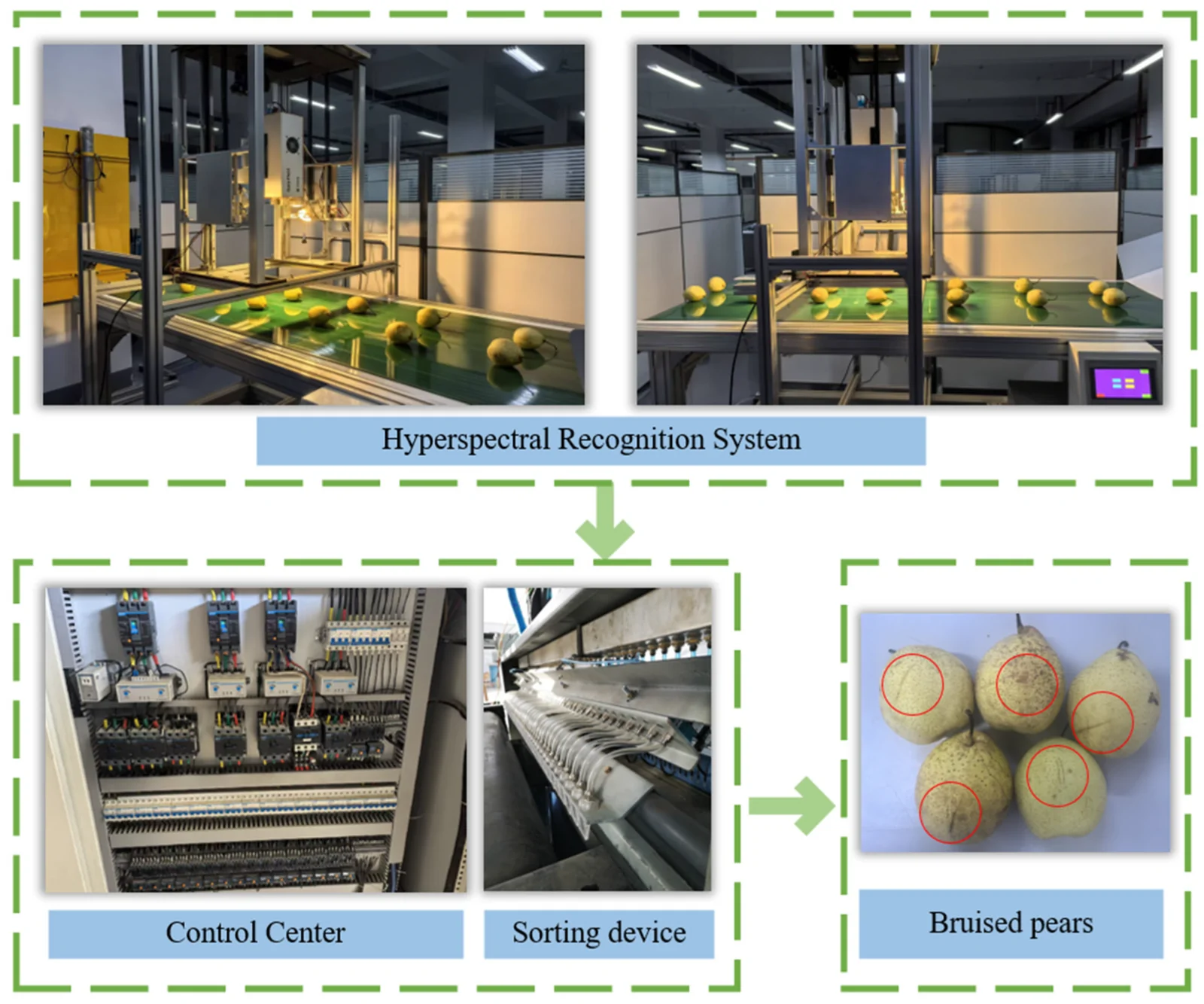
Verification Process for Hyperspectral Sorting Trials.

To further validate the model’s performance in real-world fruit-level sorting scenarios, a further 300 independent Yali pear samples (150 healthy fruits and 150 damaged fruits) were selected specifically for fruit-level sorting validation; these samples did not overlap with those used for model training. Figure 12 illustrates the validation workflow for the hyperspectral sorting trial. The damaged samples comprised two typical types of mechanical damage: Mechanical scratch and Compression damage. None of the fruit-level validation samples were used in the aforementioned pixel-level model training, parameter optimisation or testing processes; they were used solely for the final independent validation.

During the practical sorting validation phase, three different model combinations were used to perform pixel-level predictions on the hyperspectral images of each fruit. As the practical sorting task primarily focuses on whether the fruit has sustained mechanical damage, the ‘Mechanical scratch’ and ‘Compression damage’ pixels output by the models were consolidated into a single ‘damaged’ pixel category, whilst ‘intact tissue’ pixels were treated as ‘healthy tissue’ pixels; background pixels were excluded from the calculation of the fruit-level damage proportion.

For a single Yali pear sample, let *N_s_* and *N_c_* be the numbers of pixels within the fruit region predicted to be affected by Mechanical scratch and Compression damage, respectively, and let *N_f_* be the total number of valid pixels in the fruit region; the proportion of damaged pixels, *R_d_*, is defined as in Equation (11).

(11) $$R_{d}=\frac{N_{s}+N_{c}}{N_{f}}\;\times \;100\%$$

When *R_d_* exceeds the preset threshold *T*, the fruit is classified as damaged; otherwise, it is classified as healthy. To avoid bias in the results caused by adjusting the threshold on an independent test sample, this paper determines the fruit-level damage classification threshold based on the validation set partitioned during the training phase. Specifically, the damage pixel ratio threshold T is scanned across the validation set in 0.1% increments, and the threshold corresponding to the maximum F1 score is adopted as the final threshold. Once the threshold has been determined, it is fixed for use in the practical sorting validation of 300 independent fruit samples.

**Figure 13:**
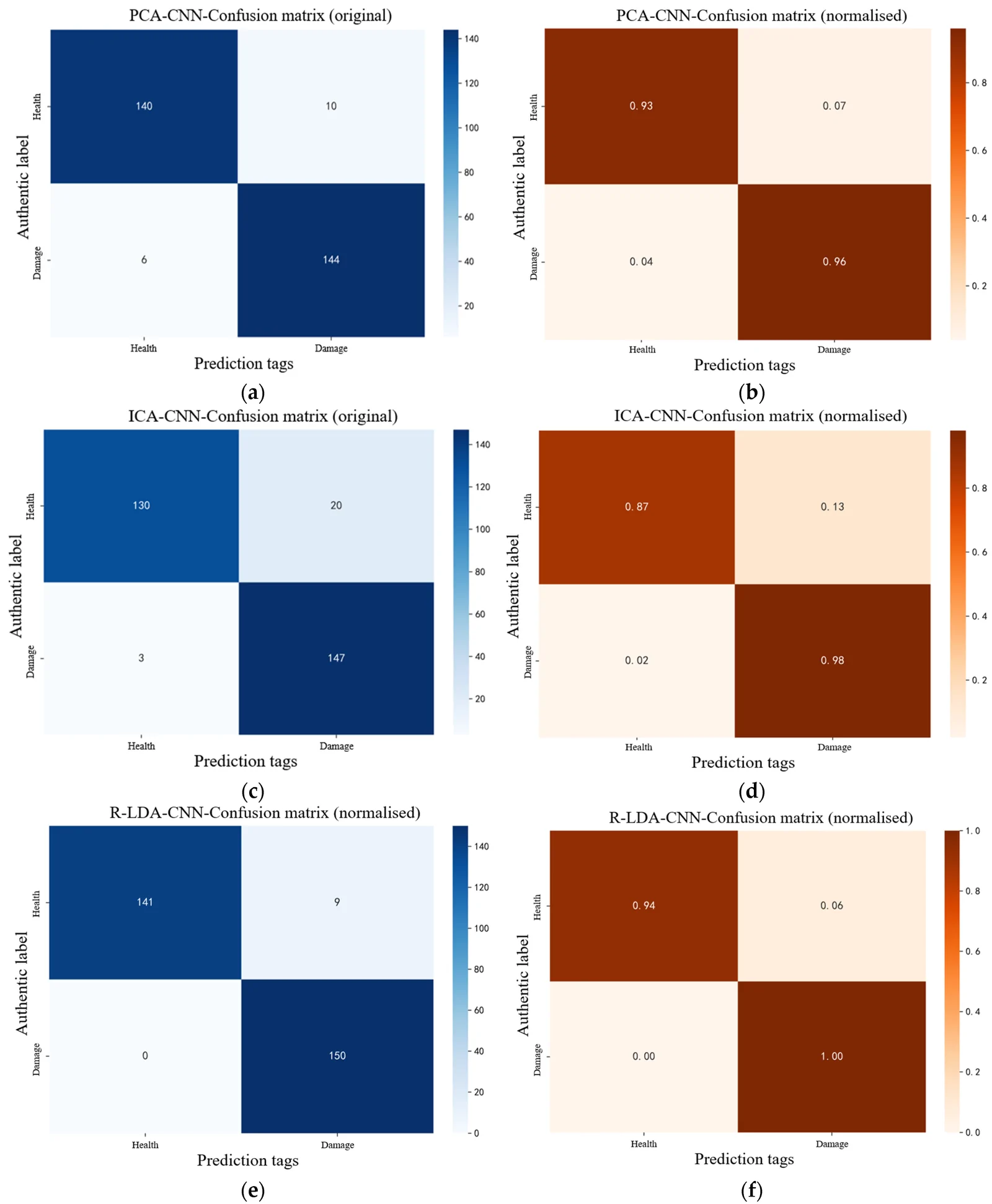
Confusion matrices for fruit-level health or damage binary classification using different feature extraction strategies combined with a lightweight CNN: (**a**) PCA-CNN-Confusion matrix (original); (**b**) PCA-CNN-Confusion matrix (normalised); (**c**) ICA-CNN-Confusion matrix (original); (**d**) ICA-CNN-Confusion matrix (normalised); (**e**) R-LDA-CNN-Confusion matrix (original); (**f**) R-LDA-CNN-Confusion matrix (normalised).

As shown in Figure 13, there are marked differences in the error distributions of the three feature extraction strategies combined with a lightweight CNN in the fruit-level binary classification task. The rows of the confusion matrix represent the actual classes, the columns represent the model’s predicted classes, the elements on the main diagonal indicate the number of correctly classified samples, and the off-diagonal elements indicate the number of misclassified samples. The confusion matrix data for the fruit-level binary classification using the three feature extraction strategies combined with a lightweight CNN are shown in Table 9. Among the independent validation samples comprising 150 healthy fruits and 150 damaged fruits, the PCA + lightweight CNN correctly identified 140 healthy fruits and 144 damaged fruits; however, 10 healthy fruits were still misclassified as damaged, and 6 damaged fruits were misclassified as healthy. ICA combined with a lightweight CNN correctly identified 147 damaged fruits, reducing the number of missed damaged fruits compared to the PCA approach; however, the number of healthy fruits misclassified as damaged increased to 20, indicating that whilst this method improves the detection rate of damaged fruits, it reduces the ability to retain healthy fruits. In contrast, the R-LDA + lightweight CNN correctly identified 141 healthy fruits and all 150 damaged fruits, with no missed detections of damaged fruits and only 9 healthy fruits misclassified as damaged. These results indicate that the supervised discriminative features obtained after R-LDA pre-processing can more effectively support the lightweight CNN in fruit-level health/damage classification, performing particularly well in reducing the risk of missed detections of damaged fruits. In practical post-harvest sorting scenarios, the failure to detect damaged fruit may result in damaged samples being mixed into batches of marketable fruit. Therefore, the fact that the R-LDA combined with a lightweight CNN ensures the complete detection of damaged fruit whilst maintaining a high recognition rate for healthy fruit suggests that this approach is more suitable for the practical sorting and inspection of mechanically damaged Yali pears.

The results of fruit-level classification were evaluated using accuracy, recall, specificity, F1 score and confusion matrices; the results are shown in Table 10. All three feature extraction strategies, when combined with a lightweight CNN, demonstrated a certain level of sorting capability in the fruit-level health/damage binary classification task. R-LDA combined with a lightweight CNN achieved the best sorting performance, with a mean accuracy of 97.00% (95% CI: 95.80–98.20%), which was higher than the 94.67% (95% CI: 93.17–96.17%) achieved by PCA combined with a lightweight CNN and the 92.33% (95% CI: 90.53–94.13%) achieved by ICA combined with a lightweight CNN.

In terms of the ability to detect damaged fruit, the recall rate of R-LDA combined with a lightweight CNN reached 100.00 per cent, meaning that none of the 150 damaged Yali pears were missed; the recall rate for PCA combined with a lightweight CNN was 96.00 per cent (6 missed cases), whilst that for ICA combined with a lightweight CNN was 98.00 per cent (3 missed cases). R-LDA combined with the lightweight CNN achieved zero missed detections (100 per cent recall) in all five replicate experiments. This is attributed to the high sensitivity of R-LDA’s supervised discriminative features to the spectral signals of Compression damage and Mechanical scratch, as well as the effective decision threshold provided by the voting mechanism based on the proportion of damaged pixels per fruit. In practical post-harvest sorting scenarios, missed detection of damaged fruit directly leads to defective products being mixed into commercial batches, thereby affecting overall quality; consequently, the absolute advantage of R-LDA + Lightweight CNN in terms of recall (zero missed detections) holds clear practical value. In terms of the ability to retain sound fruit, the specificity of R-LDA + lightweight CNN was 94.00% (95% CI: 92.62–95.38%), meaning that 9 out of every 150 sound fruits were misclassified as damaged; the specificity of PCA + lightweight CNN was 93.33%, whilst that of ICA + lightweight CNN was 86.67%. The latter has a false rejection rate for healthy fruit as high as 13.33% (20/150), which can easily lead to unnecessary commercial wastage, whereas R-LDA combined with the lightweight CNN achieves zero false negatives whilst maintaining a specificity of over 94%, thereby striking a better balance between recall and specificity.

Taking all four metrics into account, the F1 score for R-LDA + lightweight CNN was 97.09% (95% CI: 96.27–97.91%), higher than the 94.74% (95% CI: 93.32–96.16%) and that of ICA + lightweight CNN (92.74% (95% CI: 91.19–94.29%)). Furthermore, the confidence intervals for all metrics were the narrowest, indicating that the supervised discriminative features from R-LDA endow the model with excellent robustness to random initialization. PCA retains only the direction with the greatest global variance and thus struggles to fully capture the discriminative spectral variations associated with damage categories; whilst ICA extracts statistically independent components, it does not utilise class label information, meaning that the extracted features may contain components unrelated to damage discrimination, resulting in insufficient specificity and overall stability. In summary, the R-LDA + lightweight CNN outperforms the comparison methods in terms of damaged fruit detection rate, healthy fruit retention capability and sorting stability, and is therefore more suitable for the practical post-harvest non-destructive sorting and detection of mechanical damage in Yali pears.

## 4. Conclusions

To address the challenge of rapid, non-destructive identification of mechanical scratch and compression damage in Yali pears, this paper proposes a detection method that integrates short-wave infrared hyperspectral imaging, discriminant feature dimensionality reduction and a lightweight convolutional neural network (CNN). A complete technical workflow has been established, ranging from image acquisition and spectral pre-processing to feature dimensionality reduction, pixel-level classification and fruit-level sorting. The main conclusions are as follows:

1. Effectiveness of R-LDA for discriminant feature dimensionality reduction

The experimental results show that, based on uniform PCA pre-processing to eliminate multicollinearity, R-LDA reduces the 8-dimensional PCA principal components to a 3-dimensional discriminant feature space; when combined with a lightweight CNN, the pixel-level classification accuracy reaches 97.98% (95% CI [97.30%, 98.66%]), whilst the F1 score across five replicate experiments was 97.09 ± 0.62%, outperforming both PCA (accuracy 95.70 ± 1.18%, F1 score of 94.74 ± 1.08%). Furthermore, the confidence intervals for all performance metrics of R-LDA were the narrowest, confirming its excellent stability under random initialization conditions. The fundamental reason for R-LDA’s performance advantage lies in its ability to stably extract the most discriminative spectral features under high-dimensional, small-sample conditions through the synergistic effect of supervised learning and dual regularisation. PCA pre-processing compresses the raw spectra into principal components that are orthogonal and uncorrelated with one another, thereby eliminating collinearity between bands; the truncation mechanism of the SVD solver improves the singularity conditions of the within-class divergence matrix, enabling the projection directions to maximise between-class divergence and minimise within-class divergence whilst exhibiting greater robustness to a limited number of training samples. In contrast, PCA and ICA retain only the directions with the greatest global variance or statistically independent components, and are unable to capture such subtle damage-specific differences. Xiong et al. (2018) utilised hyperspectral data combined with the PLS-DA algorithm to achieve only a binary classification of intact versus damaged lychees, with an average recall of 94.10% and an average precision of 93.95% on the external validation set; furthermore, they were unable to distinguish between different types of mechanical damage [24]. With the same R-LDA-dimension-reduced features as input, the classification accuracy of the lightweight CNN improved by more than 6 percentage points compared to traditional classifiers such as SVM, Random Forest and PLS-DA, further validating the rationality of the design of this method. The R-LDA-CNN approach proposed in this study demonstrates significant advantages in terms of feature separability and classification precision, indicating that the introduction of class-labelled-guided dimensionality reduction is crucial for the simplification of hyperspectral data and the extraction of discriminative information.

2. Comparison and Interpretation of Damage Recognition Performance

At the pixel-level recognition stage, the recognition accuracy for Compression damage (98.60%) was higher than that for Mechanical scratch (93.54%). This difference has clear biological and physical foundations: compression damage typically causes extensive browning of subcutaneous tissue, triggering significant chemical changes such as water loss, phenolic oxidation and pigment degradation, which result in characteristic absorption responses in the near-infrared band, whereas mechanical scratches are merely superficial linear lesions, with relatively weak spectral responses that are easily affected by fruit peel curvature and uneven illumination. Nevertheless, the recognition rate for mechanical scratch remains at a high level, indicating that the lightweight CNN successfully captures subtle spatio-spectral texture features of the scratched areas through hierarchical convolutions, rather than relying solely on single-point spectral responses. Ma et al. relied solely on one-dimensional near-infrared spectra to perform binary classification of kiwifruit damage, and were unable to distinguish between different forms of mechanical damage [25]; the present study covers both types of damage—Compression damage and Mechanical scratch—and demonstrates more balanced recognition performance across these categories.

3. Significance and limitations of fruit-level validation

In a validation study involving 300 individual fruits using fruit-level sorting, the R-LDA-CNN model achieved a sorting accuracy of 97.00% (95% CI [95.80%, 98.20%]) and an F1 score of 97.09% (95% CI [96.27%, 97.91%]), with a recall rate of 100.00%—meaning no missed detections among the 150 damaged fruits—effectively eliminating the risk of damaged fruits entering commercial batches. The key to this result lies in the fact that the R-LDA-CNN’s precise, pixel-level activation of damaged regions ensures zero missed detections, which is then reliably mapped to the fruit-level label via a voting mechanism based on the proportion of damaged pixels within each individual fruit. The transition of the classification unit from ‘pixels’ to ‘fruits’ validates the method’s potential for practical application on online sorting lines. It should also be noted that the current false positive rate in fruit-level validation is approximately 6 per cent, with some healthy fruits (9 out of 150) being misclassified as damaged. In future, the false rejection rate could be further reduced by optimising the sorting threshold strategy or introducing multi-view imaging.

Under the experimental conditions of this study, the proposed method demonstrated good performance in identifying mechanical damage in the target fruit and shows considerable potential for further research and practical application. However, this study still has the following limitations: firstly, it has not yet been validated across different varieties, stages of ripeness or harvesting seasons, nor has the influence of storage conditions and the duration of post-damage detection been investigated; secondly, the damaged samples were artificially prepared in the laboratory and may differ from naturally occurring damage in terms of mechanical causes and physiological changes; thirdly, although the fruit-level validation samples were entirely independent of the modelling fruits, they were still sourced from the same orchard during the same period and therefore do not constitute external validation across different orchards or seasons. Future work will further validate the model’s generalization ability across different varieties, ripeness levels, damage times, naturally damaged samples and online sorting conditions, thereby advancing its practical application.

## Figures and Tables

**Table 1:** ROI annotation rules and sample sizes for each category.

Category	ROI Source Rules	Total Number of ROIs	Average Number of Pixels per ROI
Intact tissue	For each healthy fruit, annotate 3 areas; for each damaged fruit, annotate 2 healthy areas	420	Approx. 156 pixels
Compression damage	For each fruit with Compression damage, annotate 1 core damaged area	60	Approx. 43.5 pixels
Mechanical scratch	For each fruit with a scratch, annotate 1 continuous core damaged area	60	Approx. 19.8 pixels
Background	For each image, annotate 4 background areas at the edges and corners	720	Approx. 117 pixels

**Table 2:** ROI: Proportion of independent data segmentation at fruit level and fruit composition.

Dataset	Percentage/%	Total Number of Fruits	Breakdown by Category
Training set	56	101	Health: 34, Mechanical scratches: 33, Compression damage: 34
Validation set	14	25	Health: 8, Mechanical scratches: 9, Compression damage: 8
Test set	30	54	Health: 18, Mechanical scratches: 18, Compression damage: 18

Note: The training, validation and test sets are divided with ‘fruit’ as the smallest independent unit. All spectral pixels and derived image tile data for a single fruit are assigned in their entirety and exclusively to the same set, thereby eliminating pixel-level pseudo-duplication of biological samples.

**Table 3:** Pixel distribution by category.

Category	Training Set ROI	Validation Set ROI	Test Set ROI	Number of Pixels in the Training Set	Number of Pixels in the Validation Set	Number of Pixels in the Test Set
Intact tissue	236	58	126	36,691	9173	19,656
Compression damage	34	8	18	1462	365	783
Mechanical scratch	33	9	18	665	166	356
Background	404	100	216	47,196	11,799	25,285
Total	707	175	378	86,014	21,503	46,080

**Table 4:** A Comparison of Feature Separation Performance Across Different Feature Extraction Strategies.

Method	Type	Fisher’s Criterion (J)	Separation Index	Computation Time (s)
PCA	Unsupervised	2.8694	2.0064	1.2837
ICA	Unsupervised	0.4326	1.3530	3.9048
R-LDA	Supervised	5.0275	2.3543	2.9149

**Table 5:** Detailed parameter configurations for each module of a convolutional neural network.

Module	Layer Types	Number of Filters	Nucleus Size	Output Dimensions	Regularisation/Notes
Input layer	Input	—	—	5 × 5 × d	d = 8 (PCA-CNN and ICA-CNN) or d = 3 (R-LDA-CNN)
Convolution block 1	Conv×2	48	3 × 3/1	5 × 5 × 48	L2 (λ = 1 × 10^−4^); BN + ReLU
	MaxPool	—	2 × 2/2	2 × 2 × 48	—
	Dropout	—	—	2 × 2 × 48	*p* = 0.2
Convolution block 2	Conv×2	96	3 × 3/1	2 × 2 × 96	L2; BN + ReLU
	MaxPool	—	2 × 2/2	1 × 1 × 96	—
	Dropout	—	—	1 × 1 × 96	*p* = 0.3
Convolution block 3	Conv×3	192	3 × 3/1	1 × 1 × 192	L2; BN + ReLU
	Dropout	—	—	1 × 1 × 192	*p* = 0.4
Classification module	GlobalAvgPool	—	—	192	Global average pooling, outputting a 1 × 1 × 192 feature vector
	Dense	128	—	128	L2; BN + ReLU; dropout (*p* = 0.5)
	Softmax	4	—	4	Outputs classification probabilities for four classes

Note: The three feature extraction strategies share exactly the same CNN architecture; only the number of channels d in the input layer is adapted according to the feature dimension—the input for PCA-CNN and ICA-CNN is 5 × 5 × 8 (d = 8), whilst that for R-LDA-CNN is 5 × 5 × 3 (d = 3). The parameters of all other layers are identical across the three models.

**Table 6:** Pixel-level classification performance of different classifiers using R-LDA-dimension-reduced features.

Classifiers	Input Format	Accuracy/%
SVM (RBF kernel)	Flatten 3D feature vectors	91.25
Random Forest	Flatten 3D feature vectors	89.80
PLS-DA	Flatten 3D feature vectors	90.52
Lightweight CNN	5 × 5 × 3 spatio-spectral image patches	97.98

**Table 7:** Comparison of pixel-level classification performance for lightweight CNNs combined with different dimension reduction methods.

Combination	Accuracy/%	Loss	Epochs
PCA + lightweight CNN	95.70 ± 1.18	0.070 ± 0.016	30
ICA + lightweight CNN	95.10 ± 1.32	0.080 ± 0.018	30
R-LDA + lightweight CNN	97.98 ± 0.68	0.040 ± 0.010	30

Note: Performance metrics are reported as ‘mean ± half-width of the 95 per cent confidence interval’. Given the limited number of replicates (n = 5), the confidence interval was calculated using a t-distribution (95 per cent CI = mean ± 2.776 × standard error). The total pixel spectral data were derived from 180 independently modelled fruits. The independent biological replicates were fruits (n = 180), not pixels.

**Table 8:** Performance by category for pixel-level classification using R-LDA+ and a lightweight CNN.

Category	Precision (%)	Recall (%)	F1 Score (%)
Intact tissue	99.08	96.33	97.69
Compression damage	72.42	98.60	83.52
Mechanical scratch	56.35	93.54	70.34
Background	99.22	99.32	99.27
Macro-F1	—	—	87.71
Balanced Accuracy	—	—	96.95

**Table 9:** Confusion matrix for fruit-level binary classification combining different feature extraction strategies with a lightweight CNN.

Model Portfolio	Actual Health Forecast Health TN	Actual Health Prediction Error FP	Prediction of Actual Damage: Healthy FN	Actual Damage vs. Predicted Damage (TP)
PCA + Lightweight CNN	140	10	6	144
ICA + Lightweight CNN	130	20	3	147
R-LDA + Lightweight CNN	141	9	0	150

Note: TN refers to the number of healthy fruits correctly classified as healthy; FP refers to the number of healthy fruits incorrectly classified as damaged; FN refers to the number of damaged fruits incorrectly classified as healthy; and TP refers to the number of damaged fruits correctly classified as damaged.

**Table 10:** A Comparison of Fruit-Level Sorting Performance Using Different Feature Extraction Strategies Combined with a Lightweight CNN.

Model Portfolio	Accuracy Rate/%	Recall Rate/%	Specificity/%	F1 Value
PCA + Lightweight CNN	94.67 ± 1.50	96.00 ± 1.35	93.33 ± 1.60	94.74 ± 1.42
ICA + Lightweight CNN	92.33 ± 1.80	98.00 ± 1.05	86.67 ± 2.00	92.74 ± 1.55
R-LDA + Lightweight CNN	97.00 ± 1.20	100.00 ± 0.00	94.00 ± 1.38	97.09 ± 0.82

Note: Values are reported as ‘mean ± half-width of the 95 per cent confidence interval’. The results are based on five independent replicates using different random seeds. The means shown in the table represent the average of the five experiments; Table 9 presents the confusion matrix results from one representative experiment, with all metrics falling within the confidence intervals reported in this table.

## Data Availability

The original contributions presented in this study are included in the article. Further inquiries can be directed to the corresponding author.
